# Supporting physical education teachers to create an empowering motivational climate

**DOI:** 10.3389/fpsyg.2026.1771885

**Published:** 2026-05-15

**Authors:** Stéphanie Girard, Audrey-Anne de Guise, Élise Désilets, Jean-François Desbiens, David Bezeau

**Affiliations:** 1 Department of Physical Activity Sciences, Université du Québec à Trois-Rivières, Trois-Rivières, QC, Canada; 2 Faculty of Physical Activity Sciences, Université de Sherbrooke, Sherbrooke, QC, Canada

**Keywords:** educational consultant, partnership-based approach, professional development, research action, teacher training

## Abstract

**Introduction:**

To support PE teachers in sustaining students’ motivation, in-service training is a promising avenue to help them create an empowering motivational climate. A training was developed and evaluated in the province of Quebec (Canada) to support PE teachers in the implementation of empowering motivational strategies. A 2-year follow-up support was offered to PE teachers by their educational consultant (EC). This collaborative study aimed to examine the outcomes of this support on the observed motivational climate and on pupils’ perceptions of the motivational climate, basic psychological needs satisfaction, achievement goals, motivation, effort and intention to be physically active.

**Method:**

A research-action design was used. The expected heterogeneity involved in implementing support modalities called for a comparison group (CG; PE teachers who did not follow the training or receive support; *n* = 5). The experimental group (EG) consisted of 9 PE teachers supported by 6 ECs. A total of 130 videos were analyzed with systematic observations and 329 pupils in the EG and 166 pupils in the CG completed self-reported questionnaires. Because of the large number of potential comparisons across groups and time periods, a bootstrap approach with confidence intervals was privileged over multiple hypothesis testing.

**Results:**

Only few changes were observed on pupils’ motivational variables between the beginning and end of each year and changes in pupils’ perceptions did not differ between groups. PE teachers in the EG were, overall, less need-thwarting at the end of each year. Moreover, need-support from teachers in the EG appears to be higher than those in the CG.

**Discussion:**

This project proved a highly enriching experience for both practitioners and researchers. Both parties recognized the need to continue efforts over a longer period in view of the uncertainties inherent in working to support the development of teachers’ professional competencies and thereby promote pupils’ motivation and engagement toward PE. The study underscores the necessity of working in collaboration with ECs to assist them in appropriating theoretical content and developing teacher support.

## Introduction

Children and teenagers are not sufficiently active to benefit from physical activity practice (World Health Organization, 2022; ParticipACTION, 2024; Valladares-Garrido et al., 2025). Physical education (PE) is a solution among others that offers young people important opportunities in this regard (Dudley et al., 2011). Sustaining pupils’ motivation toward PE is therefore essential, as it strongly relates to their engagement in PE and their participation in physical activity outside school (Huotari et al., 2011; Sierra-Diaz et al., 2019). Accordingly, an important part of the PE teacher’s role is the use of various motivating strategies to increase the quality of pupils’ engagement (Weeldenburg et al., 2022; Carriedo et al., 2023), not always an easy task, as even well-intentioned PE teachers may use ineffective or counterproductive strategies (Diloy-Peña et al., 2021; Van Doren et al., 2021; Milton et al., 2025a). Given that engagement in physical activity during childhood and adolescence predicts physical activity practice in adulthood (Huotari et al., 2011; Sierra-Diaz et al., 2019), PE teachers require, in addition to practical skills, deeper theoretical knowledge supported by scientific evidence to effectively motivate and engage their pupils (Kim, 2020).

To foster individuals’ motivation and engagement, Duda, (2013) combined features from self-determination theory (Ryan and Deci, 2000) and achievement goal theory (Ames, 1992) to underscore the relevance of creating an empowering motivational climate in the context of sport, one defined as need-supportive (support autonomy, competence, and relatedness needs) and mastery-oriented. This type of climate is conducive to an individual’s intrinsic and self-determined forms of motivation, adoption of mastery goals (i.e., strives to improve by defining success based on personal progress), well-being, and optimal functioning (Duda and Appleton, 2016; Duda et al., 2018). A disempowering motivational climate, in contrast, is defined as need-thwarting (controlling, chaotic, and relatedness-thwarting) and performance-oriented, and tends to promote an individual’s controlled forms of motivation and amotivation, adoption of performance goals [i.e., strives to outperform others (performance-approach goals) or avoidance of underperforming compared to others (performance-avoidance goals)], ill-being, and compromised functioning (Duda and Appleton, 2016; Duda et al., 2018).

Duda’s initial conceptualization (2013) evolved to include up to eight dimensions of the motivational climate (Smith et al., 2015) and has been applied in the PE context (Girard et al., 2023b; Vlachos and Papaioannou, 2023; Girard and de Guise, 2024). Table 1 describes each dimension of an empowering and a disempowering motivational climate.

**Table 1:** Dimensions of an empowering and a disempowering motivational climate.

Dimensions		Empowering motivational climate	Dimensions		Disempowering motivational climate
Autonomy-support		Provides opportunities for individuals to understand the reasons behind their behaviour and ensures they feel heard.	Control		Controls and restricts opportunities for individuals to be heard or considered.
Competence- support	Mastery (AGT)	Values progression, improvement, and effort.	Competence-thwarting	Performance (AGT)	Values superior ability and performance.
	Structure (SDT)	Provides clear and concise instructions and expectations for learning.		Chaos (SDT)	Is inconsistent, unclear and chaotic.
Relatedness-support		Creates a space for individuals to feel psychologically safe and connected with others.	Relatedness-thwarting		Limits quality relationships and establishes a space where some individuals feel psychologically unsafe.

Both motivational theories are strongly supported in the context of studies in sport (Duda et al., 2024; Martinez-Gonzalez et al., 2025) and PE (Haerens et al., 2018; García-González et al., 2019; Girard et al., 2019; Blais et al., 2020; Cents-Boonstra et al., 2020; De Meester et al., 2020; Mastagli et al., 2022; Fierro-Suero et al., 2024; Simon et al., 2025b; Milton et al., 2025b), leading researchers to focus increasingly on ways to help teachers implement these practices to encourage pupils’ motivation, enjoyment, concentration, and effort in PE (Mastagli et al., 2022; Girard et al., 2023b; Milton et al., 2025a; Simon et al., 2025b).

A particular strategy used in English-speaking Canada, the United States and Europe involves reliance on “instructional coaches” or “pedagogical advisors” (Knight, 2009) to offer teachers in-service training and support. However, the definition of this role differs across countries and regions in terms of both qualifications required and responsibilities (Devine et al., 2013; Renard and Lothaire, 2022). In French-speaking Canada (Quebec), in-service teachers’ professional development relies mainly on “educational consultants (EC)” (Guillemette, 2021), who tend to be experienced teachers interested in supporting colleagues in terms of their pedagogical practices. They therefore have no hierarchical superiority or more advanced training (although these are possibilities). In the specific context of PE, some teachers become full-time ECs, while others become an EC while maintaining their role as teachers, dedicating only a portion of their time (varying from one person to another) to the role of EC (Guertin-Wilson, 2021). They generally work with teachers from the same school board, which provides improved support owing to proximity. As a result, they serve as essential partners in teacher training. Given the nature of their role and their close connection with teachers, ECs are ideal collaborators for delivering effective in-service training (Guertin-Wilson, 2021).

### Study rationale

**Figure 1:**
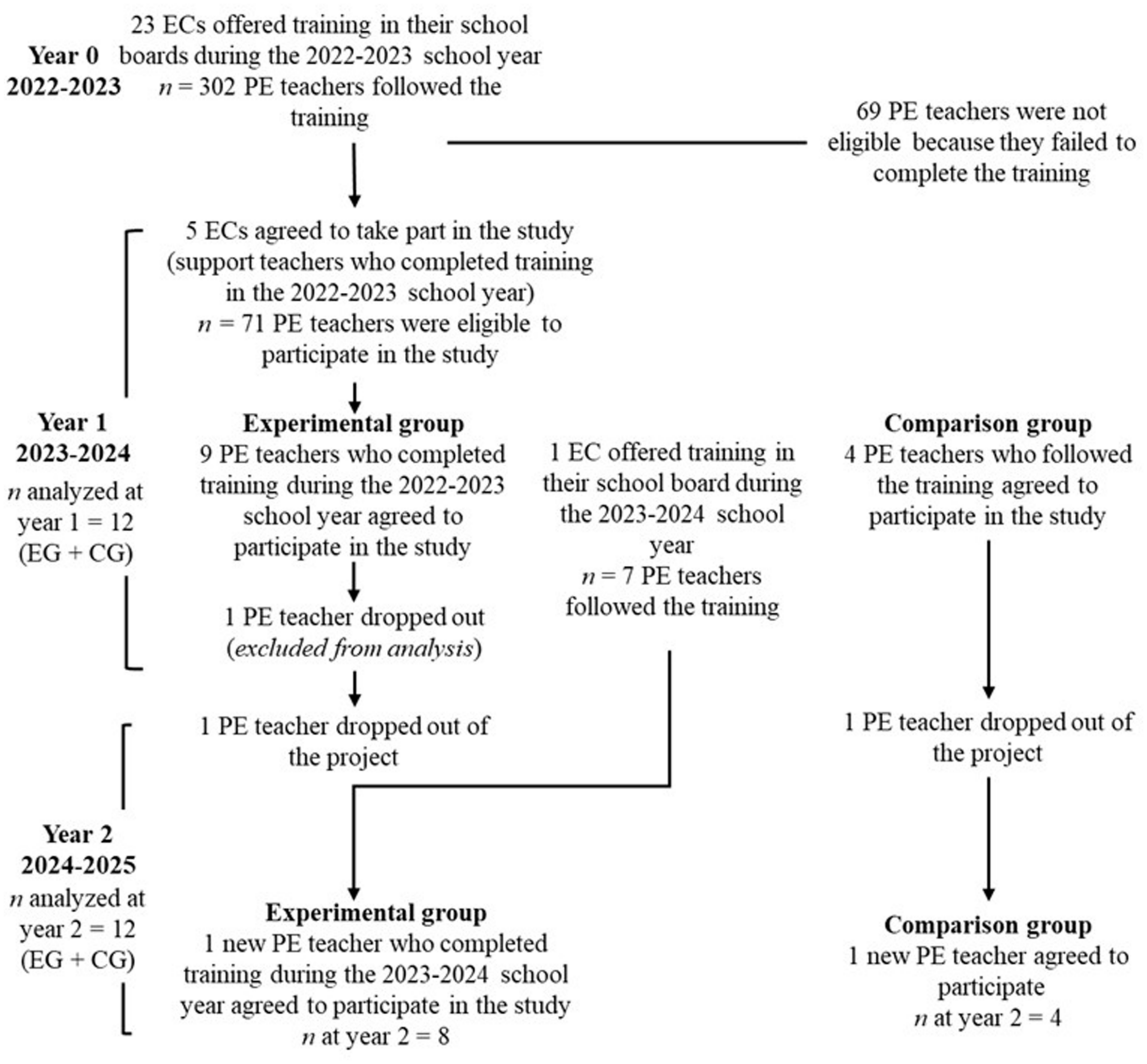
Flow chart.

In line with the above, the *Motivate to learn* training course was developed by researchers in partnership with a team of ECs in Quebec to support PE teachers in the implementation of an empowering motivational climate. The training was first delivered in the context of a pilot study (2018–2019) offered by an EC and a research team member. Given the satisfaction and appreciation of all stakeholders involved in the program’s development and subsequent to the evaluation of training in a pilot study (full version; Girard et al., 2023b), the *Fédération des éducateurs et éducatrices physique enseignants du Québec* (FEEPEQ) obtained funding from the Ministry of Education to support the training’s province-wide deployment in 2022–2023 (year 0; see flow chart in Figure 1). Because this took place during the pandemic, ECs revised, condensed, and offered the training online. The funds received also made it possible to employ two “expert” ECs to support this deployment to act as members of the research team. Generally speaking, both versions of the training (in-person [full version] and online [condensed version]) were designed with a clear, structured framework to facilitate delivery by ECs. Offered over a school year, this format aimed to ensure consistent and faithful implementation of the content provided to teachers (for a detailed description of the development of the training, see Girard et al., 2023b). All in all, 233 PE teachers (183 elementary school teachers and 50 high school teachers) from 23 school boards completed one of the two versions during the 2022–2023 school year.

Quantitative results of the evaluation of the pilot training (2018–2019) revealed that the training had no significant effect on pupils’ motivation but could effectively prevent its deterioration during the academic year (Girard et al., 2023b). However, some minor effects were observed regarding the practices of PE teachers who attended the course. These practices, to be specific, were more empowering at the end of the lesson (during the integration phase), whereas the motivational climate of the overall lesson tended to be more empowering at the end of the academic year (Girard et al., 2023b). The training program also influenced PE teachers’ beliefs about the ease of implementing learned motivational strategies, but not those about their effectiveness (Girard et al., 2023a). Overall, although the quantitative data showed mitigated results regarding changes in PE teachers’ behaviors and beliefs as well as in pupils’ perceptions of the motivational climate, the qualitative data continues to support the necessity and relevance of this training for PE teachers (Hogue, 2022). Indeed, teachers reported that the training helped them implement the strategies learned and positively impacted their pupils’ motivation and engagement (Hogue, 2022). Thus, consistent with studies by Girard et al. (2023a,b) and Milton et al. (2025a), ongoing support remains essential even after a structured and extended training program. These authors argue that, beyond the delivery of content and practice opportunities, teachers benefit from continued, individualized guidance to help them consolidate and adapt what they have learned. Support modalities such as participation in communities of practice, feedback on implemented strategies, and time and assistance with lessons have been identified as effective ways to foster sustained professional development (Armour et al., 2015; Tannehill et al., 2021). Thus, this large-scale project aimed to strengthen the support offered to PE teachers following completion of the *Motivate to learn* training course. To allow ECs to offer individualized support for implementing an empowering motivational climate in PE during the 2 years following training (Cordingley, 2015; Desimone and Garet, 2015; Darling-Hammond et al., 2017; Kyriakides et al., 2017), a Partnership Development Grant from the Social Sciences and Humanities Research Council (SSHRC 890–2021-0095) was obtained. Three partners were involved in the project, namely, two departments from the Quebec Ministry of Education^1^ and the FEEPEQ. Their contribution allowed ECs offering the training to continue supporting PE teachers for 2 years while collaborating with the research team. In sum, this study represents the culmination of a series of research initiatives conducted across three distinct phases: (1) evaluation of the pilot training (full version; 2018–2019; Girard et al., 2023a, 2023b); (2) provincial rollout of the training program (full and condensed versions; 2022–2023); and (3) the present study, which focuses specifically on the 2 years of post-training support (2023–2024 and 2024–2025).

### Study contribution

The present study addresses two issues that warrant further scholarly attention. First, previous research has shown that PE teachers have fewer opportunities for professional development than teachers of other school subjects, and that they receive less financial compensation and release time for participating in such activities (Cardina and DeNysschen, 2018). Second, thanks to the partnership-based structure of the research project, this study is innovative in that the research team, rather than substituting for the EC, positions itself as a support lever for these professionals by embedding its actions within the realities of their day-to-day practice. This ecological value is enhanced by the two-year longitudinal design, which allowed to implemented multiple support modalities on a sustained duration, as recommended in previous work (Cordingley, 2015; Desimone and Garet, 2015; Darling-Hammond et al., 2017; Kyriakides et al., 2017). This study also contributes to filling a gap in the scientific literature, as the limited number of studies currently available underscores the relatively recent scholarly attention paid to ECs’ profession within educational contexts (Vachon et al., 2021). Finally, from a methodological standpoint, focusing on both pupils’ perceptions and PE teacher pedagogical practices allows to deepen our understanding of the mechanisms underlying the implementation of an empowering motivational climate with a view to sustaining pupils’ motivation in PE.

### Objectives

In line with the above, the present study aimed to examine the outcomes of ECs’ support for PE teachers who completed the *Motivate to learn* training course in terms of the motivational climate created and pupils’ motivational perceptions. In regard to pupils, there were two specific objectives: (1) verify if changes occurred in pupils’ perceptions of the motivational climate, basic psychological needs satisfaction, achievement goals, motivation, effort and intention to be physically active between the beginning and end of both academic years; and (2) examine if changes in pupils’ perceptions differed between groups of PE teachers who received training and support and groups who did not. In regard to PE teachers, there were two additional objectives: (3) examine if the observed motivational climate established by PE teachers evolved during each academic year; and (4) verify if the observed motivational climate established by PE teachers during both academic years differed between PE teachers who received training and support and those who did not.

## Method

This study is part of a comprehensive-interpretive paradigm aimed at contributing to teachers’ professional development (Savoie-Zajc, 2017). A research-action design is used with a view to changing a specific situation to support the professional development of the individuals involved. While bridging the gap between theory and practice, this type of research makes it possible to produce new knowledge that is of interest to both researchers and actors in the field (Guay and Prud'homme, 2018; Bergeron and Bergeron, 2021).

In contrast to experimental designs, this type of research does not usually require a control group (Fortin and Gagnon, 2022). However, because of the collaborative nature of the project, which offered ECs and PE teachers considerable flexibility, the expected heterogeneity involved in implementing support modalities called for a group to serve as a point of comparison (comparison group-CG; PE teachers who did not follow the training or receive support). Indeed, the heterogeneity of the intervention could be viewed as a potential methodological weakness affecting fidelity of implementation (Small et al., 2021). In this context, the addition of a CG allowed us to verify whether, heterogeneity notwithstanding, a general or significant trend emerges or whether the absence of results can be linked to this variability. This can be a useful result for adjusting the procedure.

### Participants and procedures

A total of six ECs took part in the research project, which involved providing a two-year follow-up support (year 1 = 2023–2024; year 2 = 2024–2025) to PE teachers who had completed the one-year training (year 0). The expected sample size was calculated in line with the amount of funding granted and the high costs associated with the data collection methods envisaged (e.g., six video recordings per teacher, which involved paying for travel expenses for several research assistants throughout Quebec, a vast region to cover) in order to recruit as many participants as possible and to prioritize greater participation in the experimental group. With these considerations in mind, the goal was to recruit 10 teachers in the experimental group and four teachers in the CG. In the end, for both years, a total of 10 PE teachers from six school boards (one per EC) were recruited for the experimental group (EG; teachers who followed the training course). However, one participant could not take part in the second measurement time during the first year (and was not available during the second year) and, therefore, is not considered in the data presented. To replace this participant, another teacher was added to the sample for the second year. Table 2 presents descriptive statistics of the PE teacher sample for both years of the project.

**Table 2:** Descriptive statistics of the PE teacher sample for each group.

Participant characteristics		EG	CG
*n*	Total (*n*)	9	5
Gender	Women (*n*)	4	0
	Men (*n*)	5	5
School level	Elementary (*n*)	4	2
	High school (*n*)	5	3
Years of age	Mean (*SD*)	41.11(9.57)	38.00(8.60)
Years of experience	Min.-Max.	7–22	3–25
	Mean (*SD*)	13.33(5.43)	12.40(8.71)

In year 1, nine teachers participated in the EG (the one who dropped out during the year, and another one who dropped out at the end of the year). It is important to note that both dropouts occurred for personal reasons that were beyond the participants’ control and were unrelated to the research project. In year 2, eight participated (including the new teacher). For the CG, four teachers were involved each year, with one dropout at the end of year 1 and one new participant for the second year. The overall process and timeline of the study from initial recruitment to final follow-up, including group allocation and participant attrition, is pictured in the flow chart (Figure 1).

**Figure 2:**
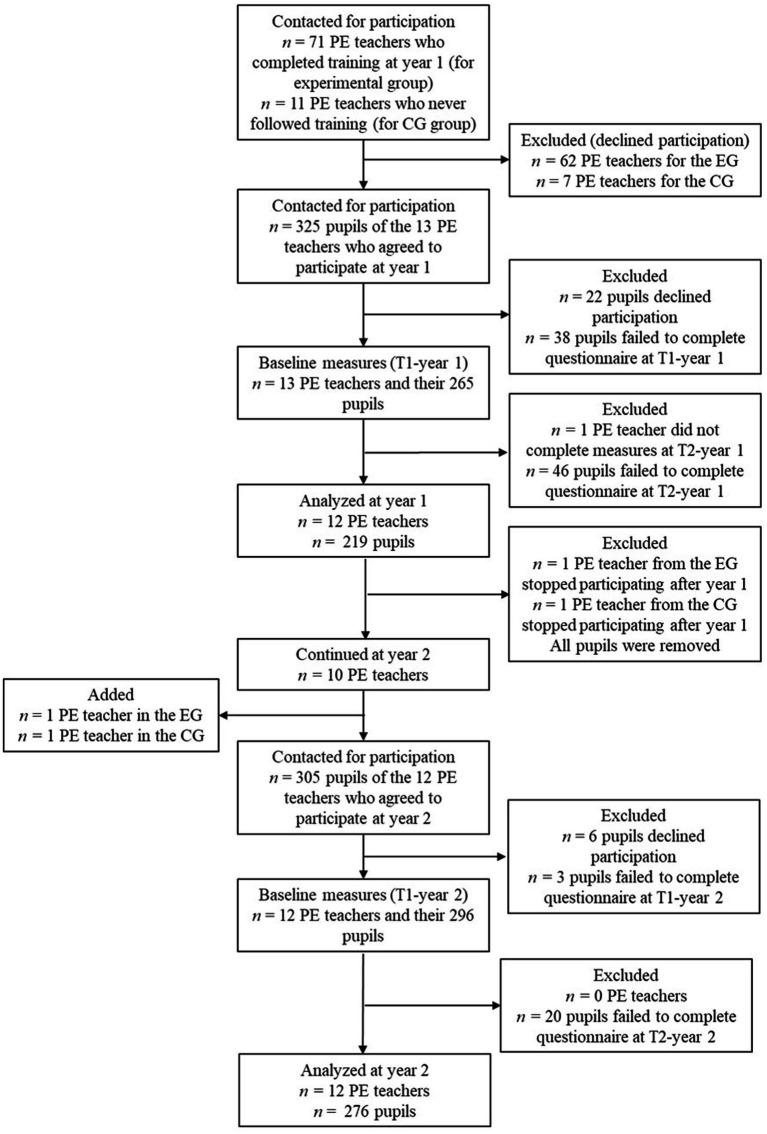
Flow diagram.

The beginning of work on the project was marked by the return to post-pandemic, in-person schooling concurrent with a teacher strike (of about 5 weeks) in Quebec, which complicated recruitment. Indeed, teachers were dealing with so much instability (e.g., pressure tactics that involved doing nothing other than the duties stipulated in their contracts, class time missed to participate in strike days, some schools closed for entire weeks, and extra work to make up for lost class time), that the thought of additional tasks for a research project was discouraging. This called for flexibility in recruitment. One strategy involved allowing teachers to choose the group of pupils they wanted to take part in the project: participation had to be beneficial for teachers in the EG and as little restrictive as possible for those in the CG. The flow diagram depicted in Figure 2 illustrates the analyzed sample showing how the number of participants decreased across stages due to withdrawals, exclusions, or incomplete data.

This study was approved by the institution’s ethics committee, and participants gave their written consent. Parents of pupils younger than 14 years old also provided their consent using an electronic form. Table 3 presents the descriptive statistics of the pupil sample analyzed each year.

**Table 3:** Descriptive statistics of the pupils sample each year.

Participant characteristics		Year 1		Year 2		Total	
		EG	CG	EG	CG	EG	CG
Gender	Girls	70	33	86	46	156	79
	Boys	73	38	92	48	165	86
	Other	4	1	3	0	7	1
School level	Elementary High school	50	39	76	46	126	85
		97	33	106	48	203	81
	Total/group	147	72	182	94	329	166
	Total/year	219		276		495	
Age (*M* in years) (*SD*)		12.5 (2.25)	12.5 (2.18)	12.7 (2.15)	12.5 (2.85)	12.6 (2.20)	12.5 (2.56)

There is one missing data for gender in the EG for year 2.

### Provision of support by EC

The governance structure of the partnership was established to enable and involve ECs in developing and updating their support modalities through participation in an implementation committee that met three times a year. The committee consisted of two researchers, two “expert” ECs who were member of the research team, all ECs who took part in the research project and one research professional. Content on the support strategies to implement with PE teachers involved the most recent recommendations regarding professional development. These could include adapting content and support methods to teachers’ specific needs, offering teachers opportunities to practice, observation (or co-observation), giving and receiving feedback, and engaging in reflective practice (Armour and Makopoulou, 2012; Guillemette, 2017; Beaudoin et al., 2018; Kraft et al., 2018; Guillemette and Monette, 2019; Makopoulou et al., 2019; Neville et al., 2020; Daigle et al., 2021; Tannehill et al., 2021; Morina et al., 2025). Different choices of tools were proposed to allow ECs to plan and document the support process they implemented each school year and were employed in different ways based on their respective contexts. The implementation committee also played a regulatory role in the project, which explains the adjustments made between years 1 and 2.

As for the PE teacher participants, funding allowed them four free half-days to benefit from EC support on the use of different modalities. In recognition of the professional autonomy and complex reality of ECs and PE teachers, they were given broad flexibility in using this time during the first year of the project. Table 4 describes the various types of follow-up support offered during both years.

**Table 4:** Description of follow-up support offered by ECs to PE teachers in the EG.

Type of follow-up support	Year 1									Year 2								
	P1	P2	P3	P4	P5	P6	P7	P8	P9	P1	P2	P3	P4	P5	P6	P7	P8	P9
Community of practice	–	–	–	**✓**	**✓**	**✓**	–	–	NA	–	–	–	–	–	–	NA	–	–
Reviewing training content with EC*	**✓**	**✓**	**✓**	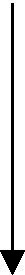	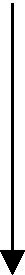	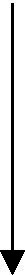	**✓**	**✓**		**✓**	**✓**	–	–	–	–		–	–
Setting personal objectives with EC*	**✓**	**✓**	–				**✓**	**✓**		**✓**	**✓**	–	–	–	–		–	–
Planning lessons based on training content with EC*	–	–	**✓**				–	–		–	–	**✓**	–	–	–		**✓**	**✓**
Time to plan lessons based on training content	–	–	–	**✓**	–	–	–	–		–	–	–	–	–	–		–	–
Observation between teachers	**✓**	**✓**	–	**✓**	–	–	–	–		**✓**	**✓**	–	–	–	–		**✓**	–
Observation of experimentation by EC	**✓**	**✓**	–	–	–	–	–	–		**✓**	**✓**	**✓**	**✓**	**✓**	**✓**		**✓**	**✓**
Feedback by EC after observations	**✓**	**✓**	–	–	–	–	–	–		**✓**	**✓**	**✓**	**✓**	**✓**	**✓**		–	**✓**
Discussion between PE teachers from different school boards	–	–	–	–	–	–	–	–		**✓**	**✓**	**✓**	**✓**	**✓**	**✓**		**✓**	**✓**

P, Participant; NA, The teacher was not included in the study during the specific year; *These support modalities were experienced in individual meetings with their EC or with small groups of teachers.

Based on feedback received from PE teacher participants at the end of the first year of support regarding their preferred support modalities and needs, as well as on discussions at the most recent implementation committee meeting with the EC, it was noted that, due to contextual situations (e.g., strike, ban on releases for any reason), some ECs were unable to provide the support they had initially planned. This being the case, two modalities were imposed for the second year of support at the first implementation meeting of this year. First, one of the four teachers’ half-day releases (pupil-free) was devoted to a meeting of all teachers in the experimental group for the purpose of sharing educational successes in creating an empowering motivational climate and discussing the challenges that remained. This meeting also aimed to offer an additional opportunity to receive support from both other teachers involved in the process and research team members. Second, ECs were asked to observe teachers in the gym and provide post-observation feedback at least once during the year (PE teachers’ preferred support modalities). This request led some ECs to express discomfort with providing on-the-spot feedback to teachers and ask the research team for support in implementing this approach.

To respond to this new need, a researcher in charge of analyzing the filmed videos enhanced the support provided to ECs outside the implementation committee while taking into account project constraints (e.g., participants were from several far-apart regions of Quebec) and ethical considerations (e.g., videos could only be viewed by the researchers responsible for analyzing them). Thus, ECs were invited to observe the PE teachers receiving support as they were being filmed for data collection. In doing so, the researcher could analyze the video prior to meeting with the EC to discuss the observations both had made during the lesson. The researcher first invited the EC to share their observations and then shared their own. The goal was to help ECs understand what could be observed, how to observe it, and what advice teachers should be given to apply the information to their practice. After this meeting, each EC met with teachers and followed the same process to share their point of view (ECs and PE teachers) regarding the lesson.

Individual meetings with ECs revealed that when they observed PE teachers using the analyzing grid provided in the training (Girard and Hogue, 2023), which consisted of 33 empowering motivational strategies (Girard et al., 2023b), ECs tended to *identify* the motivational strategies successfully implemented rather than consider ways to optimize their effectiveness or *propose other possibilities. This focus on teachers’ successes led to difficulty suggesting improvements going forward.* Based on these observations and discussions, the second implementation committee of year 2 emphasized possible disempowering strategies in PE classes, their subtle nature, and how to replace them with empowering strategies.

### Data collection

**Figure 3:**
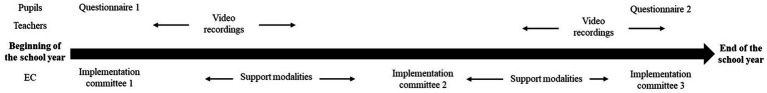
Data collection and support provision timeline.

For the two first objectives, pupils completed a questionnaire at the beginning and end of the academic year regarding their perception of the motivational climate, the satisfaction of their basic psychological needs, and their achievement goals, motivation, effort in PE and intention to be physically active. For the third and fourth objectives, one to three lessons per teacher were filmed at the beginning and end of both years of the study. They were then analyzed using an observation grid of the empowering and disempowering motivational climate (Girard et al., 2023b). Figure 3 depicts the timeline sequence of data collection, provision of support and implementation committees during both years. In short, the questionnaires were completed at different times from the video recordings, and support was provided between the two video recordings periods. The data collection periods and the provision of support extended over a prolonged period due to the challenges encountered by the schools (e.g., strikes and school closures).

### Measurement tools

#### Pupils’ questionnaires

Pupils’ self-reported questionnaires consisted of 69 items and took approximately 20 min to complete during the beginning or end of a PE class (at the teacher’s convenience). Pupils responded on a 7-point Likert scale (1 = *strongly disagree*; 7 = *strongly agree*). All scales had sufficient internal consistency (*ω* > 0.71; *α* > 0.70) with data from the sample.

#### Perceived motivational climate

This was measured using the short French version of the Motivational Climate Questionnaire-PE (SFMCQ-PE; Mastagli et al., 2026) to assess motivational climates that were empowering (6 items; e.g., *Since the beginning of the year, the PE teacher encourages pupils to help each other learn and improve*) and disempowering (6 items; e.g., *Since the beginning of the year, the PE teacher praises mostly the pupils who perform better than others*).

#### Basic psychological needs’ satisfaction

Autonomy satisfaction was measured using the autonomy questionnaire (Standage et al., 2003), which consisted of 5 items (e.g., *Since the beginning of the year, in my PE class, I feel a certain freedom of action*). Competence satisfaction was assessed using a subscale from the Intrinsic Motivation Inventory (McAuley et al., 1989), which consisted of 5 items (one reversed; e.g., *Since the beginning of the year, in my PE class, I think I am pretty good*). Relatedness satisfaction was measured using the Need for Relatedness Scale (Richer and Vallerand, 1998) consisting of 5 items (e.g., *Since the beginning of the year, with other pupils in my PE class, I feel supported*).

#### Achievement goals

The French Achievement Goals Questionnaire for Sport and Exercise (FAGQSE; Riou et al., 2012) was used to assess mastery goals (3 items; e.g., *Since the beginning of the year, in my PE class, my goal is to progress as much as possible*), performance-approach goals (e.g., *Since the beginning of the year, in my PE class, my goal is to perform better than others*) and performance-avoidance goals (3 items; e.g., *Since the beginning of the year, in my PE class, my aim is to avoid performing worse than others*).

#### Motivation

The Behavioral Regulations in Physical Education Questionnaire (BRPEQ; Aelterman et al., 2012) allowed for assessing intrinsic motivation (4 items; e.g., *I put effort in this PE class, because it is fun*), identified regulation (4 items; e.g., *I put effort in this PE class, because I find it personally meaningful*), introjected regulation (4 items; e.g., *I put effort in this PE class, because it is the only way to be proud of myself*), external regulation (4 items; e.g., *I put effort in this PE class, because I otherwise get criticized*), and amotivation (4 items; e.g., *I do not see why this PE class is part of the curriculum*). As for integrated regulation (4 items; e.g., *I put effort in this PE class, because it is consistent with my values*), it was assessed with the Behavioral Regulations in Exercise Questionnaire-III (Wilson et al., 2006).

#### Effort

This was assessed using a subscale from the Intrinsic Motivation Inventory (McAuley et al., 1989), which consisted of 4 items (one reversed; e.g., *Since the beginning of the year, in my PE class, I put a lot of effort*).

#### Intention

This was assessed with the validated French translation of the Intention to be Physically Active Scale (Dupont et al., 2009), which consisted of 5 items (e.g., *I often do sport in my free time*).

##### Observed measures

A total of 130 videos were analyzed during the 2 years of data collection. Each PE teacher from both groups (EG and CG) was filmed one to three times during one Learning and Assessment Situation at the beginning and end of years 1 and 2; Fall 2023 (EG: *n* = 20; CG: *n* = 10), Winter 2024 (EG: *n* = 22; CG: *n* = 11), Fall 2024 (EG: *n* = 23; CG: *n* = 11) and Winter 2025 (EG: *n* = 22; CG: *n* = 11). Temporal boundaries were identified for all phases of each lesson analyzed: preparation, realization, integration, and gaps, making it possible to verify the amount of time in each phase and support the interpretation of results (see Supplementary File A for detailed results). It should be noted that in Quebec, PE classes in elementary school usually last around 55 min and those in high school around 1 h and 15 min. The preparation phase consists of readying pupils for the lesson: welcoming them, stating the lesson objective, providing lesson content based on the objective, instructing them on how to apply what they learn, and doing a warm-up. This phase usually occurs at the beginning of a lesson but can be repeated throughout if teachers wish to add educational content or modify the proposed task. During the realization phase, pupils can apply the learned content in the proposed task and teachers can provide feedback and clear guidelines to help them achieve the lesson’s stated objective. In the integration phase, teachers invite pupils to reflect on their progress during the task or lesson. This usually takes place at the end of the lesson but can be used after one or more tasks as well. Finally, gaps consist of all moments between the preparation, realization, and integration phases as well as between various activities. They are periods of transition that include team creation and the management of materials and equipment.

###### Observed motivational climate

All lessons were coded using a grid in which strategies were categorized according to dimensions of the empowering and disempowering climate (Girard et al., 2023b). A total of 33 strategies were used to code teachers’ implementation of an empowering motivational climate: seven for autonomy-support (e.g., provides meaningful choice to student); eight for mastery (e.g., provides variation between or within exercises); eight for structure (e.g., demonstrates the tasks himself and/or uses pupils as a positive “role model”), and 11 for relatedness support (e.g., addresses pupils by their first name when the opportunity occurs). A total of 13 disempowering strategies were used to code the implementation of a disempowering climate: three for control (e.g., relies on authority in response to pupils’ complaints/requests); three for performance (e.g., emphasizes errors and/or performance); three for chaos (e.g., gives few or no explanations or they are imprecise); and four for relatedness-thwarting (e.g., uses sarcasm). For each lesson observed, strategies were annotated based on the phases they were used in. This provided an indicator for assigning a score for how the teacher supported and thwarted each need in each phase of the lesson: 0 = not at all; 1 = very low; 2 = low; 3 = moderately low; 4 = moderate; 5 = moderately strong; 6 = strong; 7 = very strong. Each strategy observed was noted down. When a strategy was implemented particularly well and repeated several times, the researcher took notes to indicate the strength and relevance of the action. An overall score was then assigned for each dimension of the motivational climate based on all the strategies deployed or not by the teacher. Given some strategies were more suitable for specific phases of each lesson, the score reflected what could reasonably be implemented. As an example, when assessing the implementation of the empowering motivational climate, a score of 0 was assigned when, for a given phase and dimension, the teacher deployed no empowering strategies to support the targeted need (without necessarily using disempowering strategies). A score of 3 indicated the teacher implemented a few strategies that were moderately empowering. A score of 5 indicated the teacher deployed several empowering strategies or, had use a few, but in a highly relevant manner. A score of 7 indicated the teacher consistently supported the targeted need within the dimension, using a variety of empowering strategies multiple times throughout the lesson (see Supplementary File A for detailed examples). The mean score of dimensions in each phase of the lesson was calculated resulting in 32 scores for each course [4 phases X 4 needs X 2 climates (support and thwart)]. A general score was obtained by calculating the mean of these scores but excluding scores from the gaps. Indeed, a gap is generally expected to be short; the shorter it is, the more it is considered to be effective. Thus, an “effective” gap may be neither empowering nor disempowering. Because the scores obtained during these moments require a different interpretive lens, we opted to present them separately in the results. A member of the research team proceeded to code all 130 videotaped lessons to ensure coherence analysis. To assess coding consistency, intra-rater (86%) and inter-rater (88%) reliability were calculated. To consider the proportion of agreements due to chance, Cohen’s kappa coefficient (*k*; Cohen, 1960) was calculated, and the intra-rater [*k* = 0.831; SE_(*K*)_ = 0.091; (IC: 0.661–1.001)] and inter-rater [*k* = 0.851; SE_(*K*)_ = 0.076; (IC: 0.703−0.999)] values were found to be excellent given that a *k* value between 0.81 and 1 indicates almost perfect agreement, and one between 0.6 and 0.8 indicates substantial agreement (Howell, 2009).

#### Statistical analysis

Before conducting the main analysis, missing data patterns were analyzed. Regarding teachers, we retained the data from all participants who completed a full year of support. For those who participated for only half a year (*n* = 1), their data were excluded, as our focus was the support itself rather than baseline measure alone. Consistently, only pupils who did not participate in both measurement points were excluded. For all remaining pupils, when data were missing on one of the scales at a given measurement point, pairwise deletion was applied solely to these variables. This approach was justified by the low proportion of missing data (5.84%), which met the criteria for missing completely at random (MCAR). The Mahalanobis distances were also examined to identify potential outliers among pupils. Out of a total of 495 questionnaires, only 18 participants exceeded the acceptable threshold (*p* < 0.001) based on a chi-square with *k* degrees of freedom (*k* = 26), representing 3.6% of the sample. Removing these questionnaires did not affect the results, therefore, they were retained in the analyzes.

Because of the large number of potential comparisons across groups and time periods, a bootstrap approach with confidence intervals was privileged over multiple hypothesis testing (Banjanovic and Osborne, 2016). This approach allows for strong visual interpretation of group differences and provides a robust, assumption-free method for exploring group differences while maintaining interpretability and avoiding the complications associated with multiple testing procedures (DiCiccio and Efron, 1996).

For data on both pupils and teachers, 95% confidence intervals were calculated using bootstrap resampling performed through 2000 bootstraps with replacement (DiCiccio and Efron, 1996). For each bootstrap sample, the mean was calculated to create a means distribution from which the 2.5th and 97.5th percentiles were extracted to find the 95% confidence intervals (Banjanovic and Osborne, 2016). This approach follows established guidelines to the effect that non-overlapping confidence intervals suggest meaningful differences between groups, while overlapping intervals suggest no clear evidence of difference (Payton et al., 2003). However, we acknowledge that confidence interval overlap does not provide a direct test of statistical significance, and our interpretations should be considered indicative (Schenker and Gentleman, 2001; Austin and Hux, 2002).

Cohen’s *d* effect sizes for between-group comparisons at each time point were calculated to quantify practical and pedagogical significance. To do so, Hattie (2012) suggests using *d* = 0.40 as a “hinge point” to qualify the effect, as being higher or lower than the average of interventions’ effect in education. This hinge-point is considered as a heuristic benchmark, which does not necessarily provide evidence of learning attributable to the intervention.

##### Pupils’ perceptions

To better understand the change in pupils’ perceptions, mean and confidence intervals were computed from the difference between pupils’ perceptions at the end and beginning of each year (T2 – T1). To meet the first objective, confidence intervals were compared to the 0 line: a meaningful change occurs in pupils’ perceptions at the end of the school year when confidence intervals do not cross the line. To meet the second objective, confidence intervals were compared across groups.

##### Observations of the motivational climate

Analyses of the observed motivational climate aimed to understand the extent to which PE teachers supported and thwarted each need. Mean and confidence intervals were therefore computed using the score obtained at T1 and T2 for each year. Evolution (objective 3) was interpreted by examining whether confidence intervals overlapped between T1 and T2, while group differences (objective 4) were interpreted by examining overlapping confidence intervals at each measurement time.

## Results

Changing pupil samples each year during the 2 years of support reflects the reality of school life but limits the ability to accurately assess changes in teaching practices based on pupils’ perceptions over 2 years rather than for each academic year. Similarly, the composition of each group of teachers varied from 1 year to the next. For these reasons, results will be presented and interpreted year by year.

### Pupils’ perceptions

#### Descriptive statistics

Table 5 displays descriptive statistics (mean and standard deviation) regarding pupils’ perceptions of motivational variables for each group at each measurement time for both years. In line with objective 1, means at T1 and T2 are italicized when the confidence interval for the difference does not include zero, indicating a change between the beginning and end of the academic year.

**Table 5:** Mean, standard deviation and differences between measurement times of pupils’ perceptions of motivational variables.

Motivational variables		Year 1		Year 2	
		EGMean (SD)	CGMean (SD)	EG Mean (SD)	CG Mean (SD)
Empowering climate	T1	5.43 (1.16)	5.79 (1.15)	*5.53 (1.16)*	5.84 (1.11)
	T2	5.43 (1.15)	5.70 (1.02)	*5.28 (1.28)*	5.77 (0.95)
Disempowering climate	T1	2.79 (1.26)	2.58 (1.37)	2.87 (1.30)	2.29 (1.02)
	T2	2.84 (1.17)	2.91 (1.28)	2.82 (1.48)	2.39 (1.11)
Autonomy	T1	*4.33 (1.27)*	4.60 (1.36)	4.53 (1.25)	4.41 (1.20)
	T2	*4.55 (1.16)*	4.75 (1.18)	4.48 (1.18)	4.35 (1.22)
Competence	T1	5.12 (1.35)	5.49 (1.31)	5.22 (1.27)	5.07 (1.48)
	T2	5.09 (1.26)	5.41 (1.14)	4.97 (1.37)	4.92 (1.57)
Relatedness	T1	5.04 (1.54)	5.29 (1.27)	5.08 (1.55)	5.44 (1.36)
	T2	5.11 (1.51)	5.49 (1.23)	5.05 (1.52)	5.31 (1.31)
Mastery goals	T1	5.72 (1.37)	6.13 (1.19)	*5.81 (1.36)*	5.86 (1.46)
	T2	5.70 (1.26)	6.17 (1.04)	*5.53 (1.48)*	5.89 (1.35)
Performance-approach goals	T1	2.94 (1.72)	3.01 (2.00)	3.31 (1.79)	3.16 (1.82)
	T2	3.20 (1.77)	3.39 (1.80)	3.32 (1.75)	3.30 (1.87)
Performance-avoidance goals	T1	4.08 (1.66)	4.26 (1.73)	*4.53 (1.49)*	4.54 (1.60)
	T2	4.20 (1.69)	4.56 (1.65)	*4.23 (1.61)*	4.24 (1.56)
Self-determined motivation	T1	5.06 (1.59)	5.48 (1.33)	*5.21 (1.41)*	5.22 (1.47)
	T2	5.10 (1.43)	5.67 (1.13)	*4.99 (1.55)*	5.31 (1.39)
Controlled motivation	T1	3.33 (1.14)	3.62 (1.37)	*3.33 (1.24)*	3.14 (1.23)
	T2	3.24 (1.14)	3.63 (1.32)	*3.12 (1.12)*	3.06 (1.14)
Amotivation	T1	2.43 (1.52)	2.21 (1.68)	2.33 (1.53)	*2.32 (1.57)*
	T2	2.47 (1.63)	2.38 (1.81)	2.38 (1.61)	*1.97 (1.42)*
Effort	T1	5.67 (1.23)	5.73 (1.17)	5.66 (1.20)	5.70 (1.32)
	T2	5.55 (1.23)	5.92 (1.08)	5.51 (1.24)	5.74 (1.29)
Intention	T1	*5.04 (1.63)*	5.32 (1.49)	5.16 (1.57)	5.16 (1.65)
	T2	*5.27 (1.38)*	5.41 (1.46)	5.31 (1.60)	5.11 (1.71)

**Figure 4:**
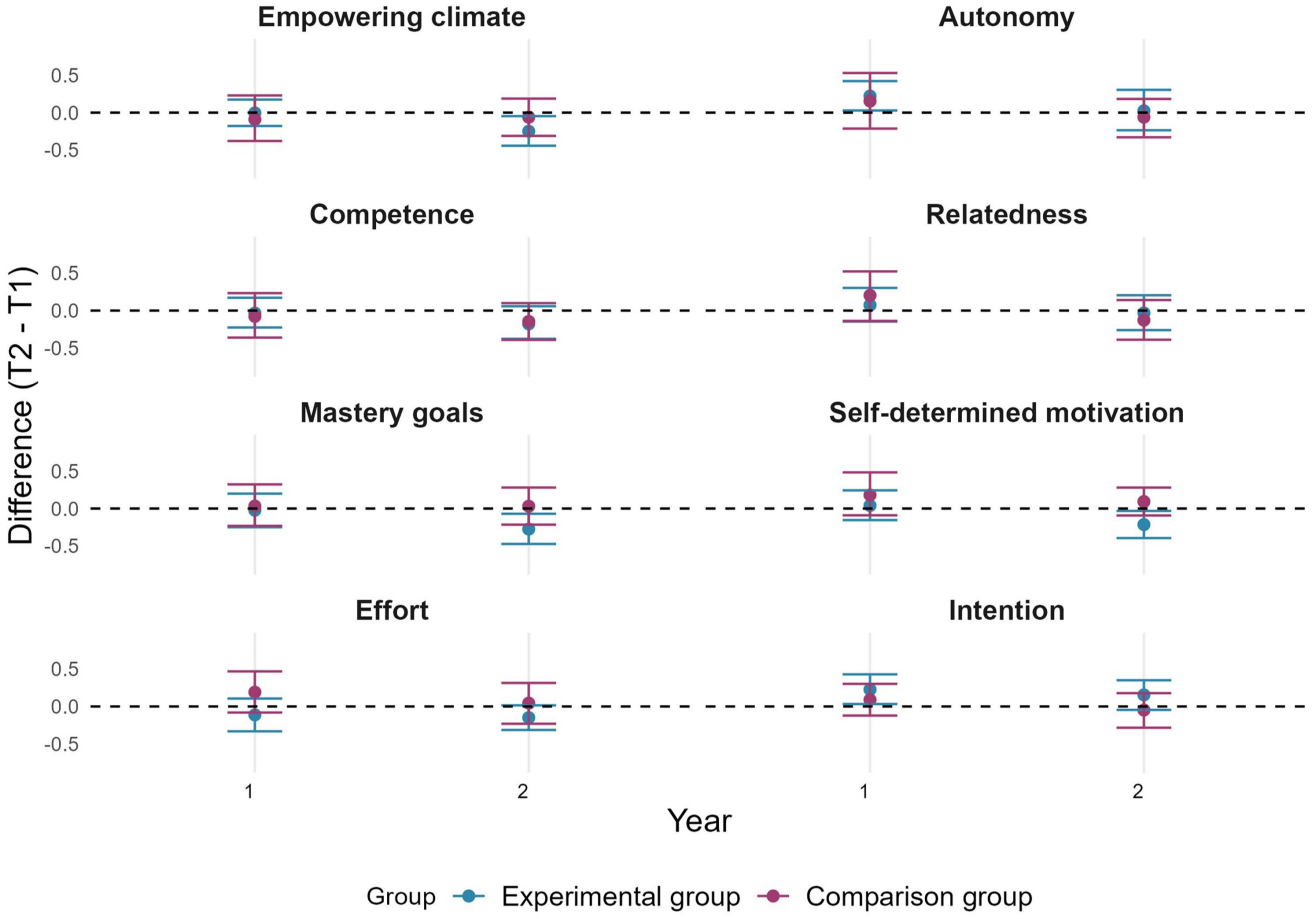
Changes in pupils’ perceptions of empowering motivational climate, basic psychological needs satisfaction, mastery goals, self-determined motivation, effort, and intention by group and by year. Error bars represent 95% confidence intervals estimated by bootstrap (*n* = 2000).

**Figure 5:**
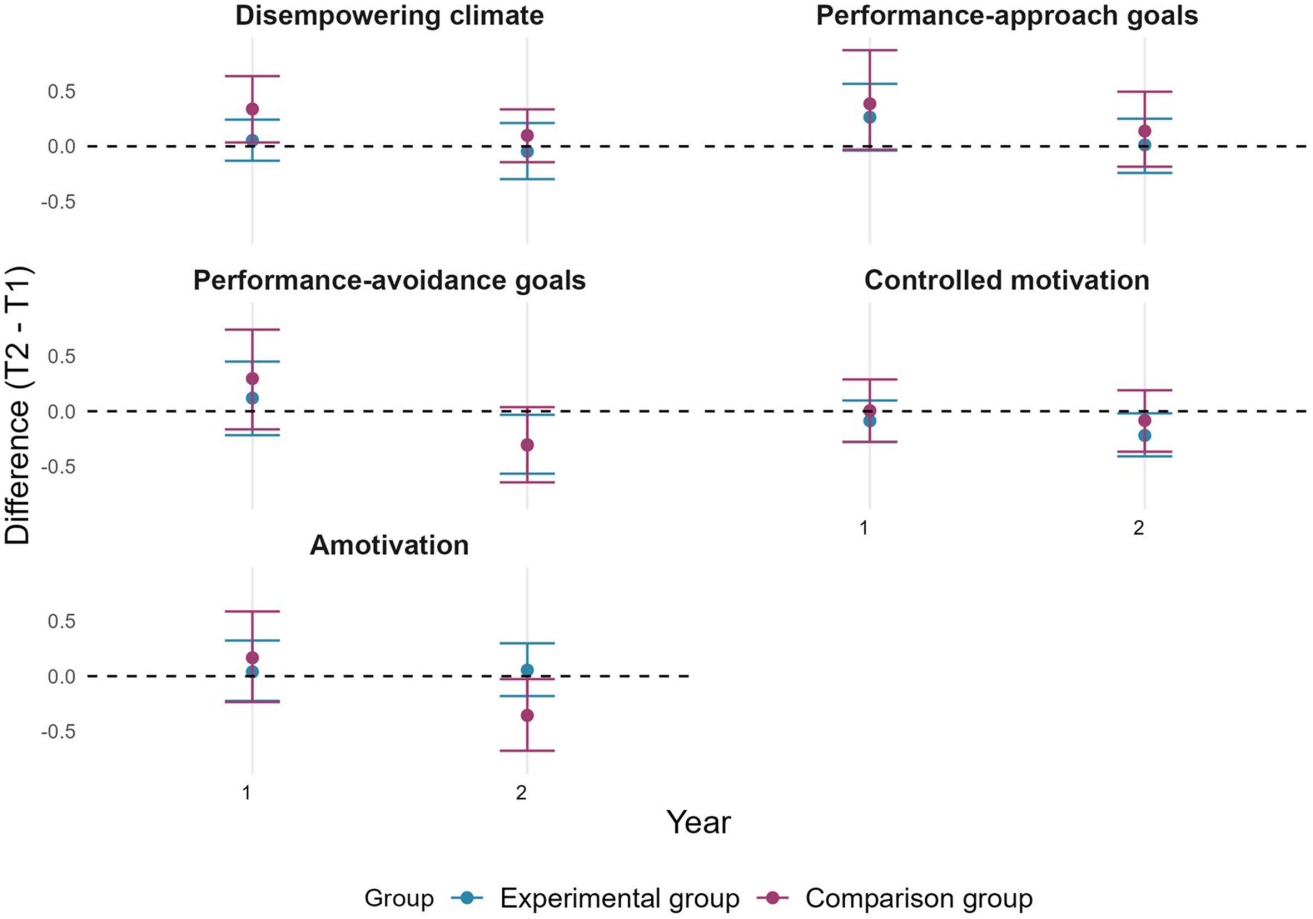
Change in pupils’ perceptions of a disempowering motivational climate, performance (approach and avoidance) goals, controlled motivation, and amotivation by group and by year. Error bars represent 95% confidence intervals estimated by bootstrap (*n* = 2000).

Italic, meaningful differences between T1 and T2 displayed in Figures 4, 5.

Overall, scores indicate that pupils from both groups are, all things considered, conducive to supporting their motivation. Indeed, scores for perceived empowering motivational climate, satisfaction of competence and relatedness needs, mastery goals, and effort are over or near 5.00/7.00. Similarly, scores for perceived disempowering motivational climate, performance-approach goals, controlled motivation and amotivation are under 4.00/7.00.

#### Objectives 1 and 2

Regarding the differences between the beginning and end of each year, although some confidence intervals overlapped, the real change remains marginal. Specifically, Figure 4 indicates that during year 1, autonomy satisfaction and intent to be physically active increased for the EG. It also shows that mastery goals, perception of an empowering climate, and self-determined motivation decreased for the EG during year 2.

Figure 5 shows that confidence intervals across time points overlap for pupils’ perceptions of a disempowering motivational climate, performance (approach and avoidance) goals, controlled motivation and amotivation during year 1. However, in year 2, controlled motivation and performance-avoidance goals decreased for the EG, while amotivation decreased for the CG.

In line with objective 2, confidence intervals of changes in pupils’ perceptions of all measured variables overlapped between the two groups for each academic year (see Figures 4, 5). Consistently, all the effects size were under 0.40. Specifically, they ranged between −0.27 and 0.26.

### Motivational climate

#### Descriptive statistics

During the first year, scores were higher overall for both groups regarding the support dimensions of an empowering motivational climate (see Supplementary File B for detailed scores). For the total lesson (excluding gaps), mean scores ranged from 2.44 to 4.48 in the EG and from 1.95 to 3.68 in the CG. For both groups, scores were higher regarding support for autonomy (total lesson). Excluding gaps, scores were generally lower in the integration phase of both groups, ranging from 0.50 to 2.90. As for the thwarting dimensions of a disempowering motivational climate, mean scores for the total lesson (excluding gaps) ranged from 0.08 to 0.91 in the EG, and from 0.97 to 1.37 in the CG. For both groups, scores were higher as regards thwarting dimensions in the realization phase, ranging from 0.27 to 2.40.

As in year 1, scores for the second year were generally higher regarding the support dimensions of an empowering motivational climate for both groups (see Supplementary File B for detailed scores). For the total lesson (excluding gaps), mean scores ranged from 1.90 to 4.08 in the EG and from 1.74 to 3.67 in the CG. Similar to year 1, scores were higher in terms of support for autonomy (total lesson), and lower in terms of competence (mastery) for both groups. As regards the thwarting dimensions of disempowering motivational climates, for the total lesson (excluding gaps), mean scores ranged from 0.13 to 1.18 for the EG, and from 0.36 to 1.27 for the CG.

#### Objectives 3 and 4

Consistent with objective 3, during year 1, PE teachers in the EG thwarted pupils’ relatedness needs less at the end of the first year during the preparation (T1 = 0.73; T2 = 0.09) and realization (T1 = 0.77; T2 = 0.09) phases, as well as during the total lesson (T1 = 0.52; T2 = 0.08). For both groups, moreover, PE teachers thwarted competence needs (structure) less during gaps at the end of the year (EG: T1 = 0.86; T2 = 0.14; CG: T1 = 0.80; T2 = 0.00). For year 2, five differences were observed among PE teachers in the EG. At the end of the year, they were less supportive of competence (structure) during the preparation phase (T1 = 5.50; T2 = 4.29) and the gaps (T1 = 2.45; T2 = 1.57). However, they also displayed less thwarting of autonomy needs during the preparation phase (T1 = 0.86; T2 = 0.14), of competence needs regarding mastery during the realization phase (T1 = 1.59; T2 = 0.55), and of competence needs regarding structure during the total lesson (T1 = 1.18; T2 = 0.46). As for the CG, both observed differences occurred during the gaps, when they demonstrated less thwarting of autonomy (T1 = 0.55; T2 = 0.00) and relatedness (T1 = 0.91; T2 = 0.00) needs. In addition to illustrating the previous stated differences (objective 3), the figures in the following sections present the results related to objective 4 in terms of the differences observed between the groups for each phase of the lesson as well as for the total lesson (excluding gaps).

#### Preparation phase

**Figure 6:**
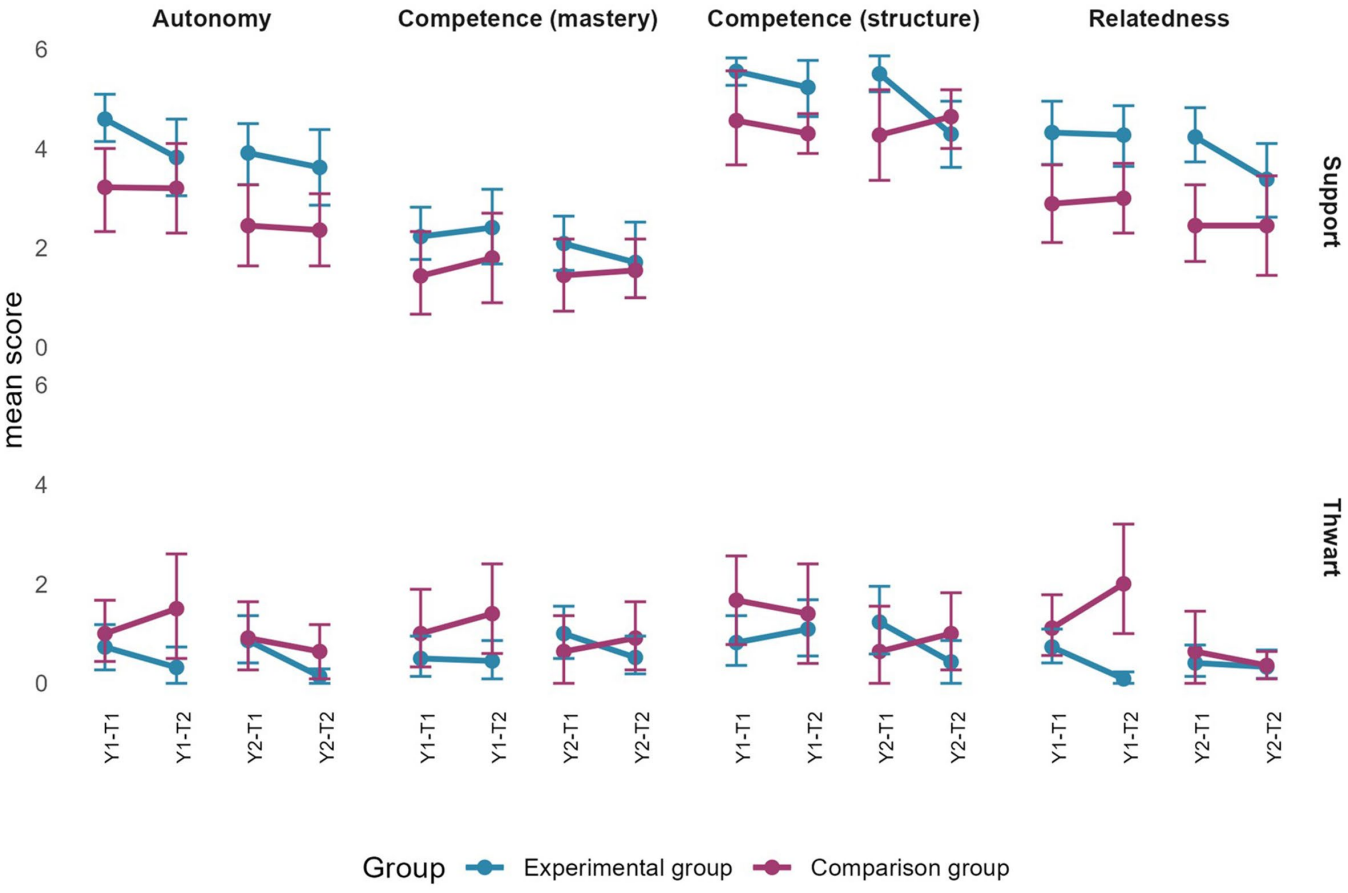
Evolution of observed motivational climate during the preparation phase between the beginning and end of each year for each group. Error bars represent 95% confidence intervals estimated by bootstrap (*n* = 2000).

The average time of the preparation phase for teachers in the EG varied between 7.92 and 27.42 min in year 1 and 9.02 and 28.15 min in year 2. For teachers in the CG, the average time in year 1 varied between 3.65 min and 18.57 min, and in year 2, between 11.78 min and 20.40 min. As Figure 6 shows, thwarting of the four needs presents some overlap between the two groups. However, at the end of the first year, teachers in the CG thwarted the need for relatedness slightly more than those in the EG. Generally speaking, teachers from the EG provided higher need-support during the preparation phase. However, the difference between the groups is meaningful only as regards autonomy-support at the beginning of the second year.

#### Realization phase

**Figure 7:**
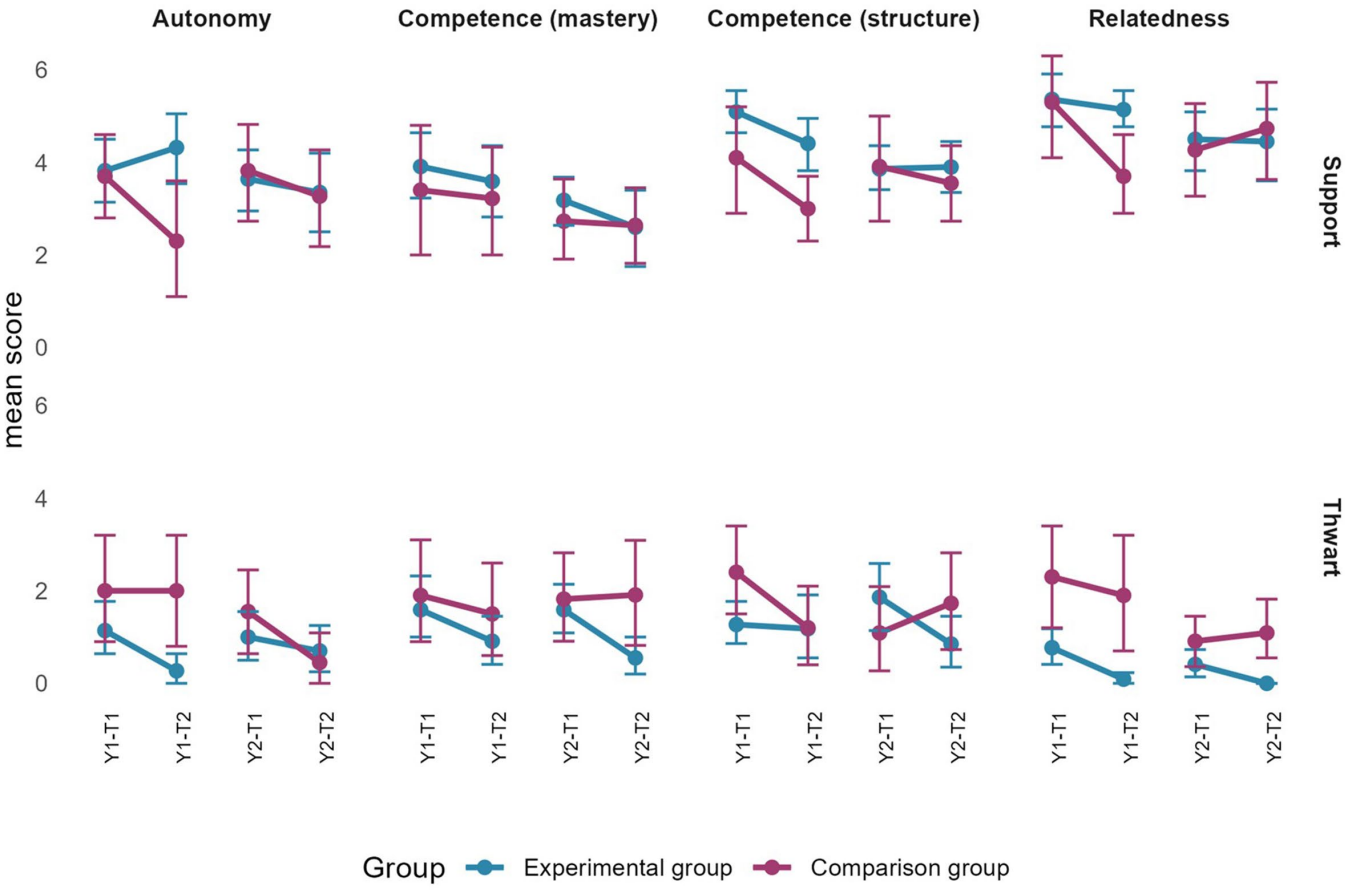
Evolution of observed motivational climate during the realization phase between the beginning and end of each year for each group. Error bars represent 95% confidence intervals estimated by bootstrap (*n* = 2000).

The average time of the realization phase for teachers in the EG varied between 20.87 and 49.47 min in year 1 and between 8.43 and 48.60 in year 2. For those in the CG, the average time in year 1 varied between 25.12 and 57.35 min, and in year 2, between 25.87 and 44.53 min. Figure 7 shows the results for the realization phase. It can be observed that teachers in the CG thwart the need for relatedness more than those in the EG at the beginning and end of the first year, as well as at the end of the second year. Autonomy-thwarting is also higher by teachers in the CG, but only at the end of the first year. As for need-support, differences between groups are visible only at the end of the first year: competence-support through structure and relatedness support is higher in the EG.

#### Integration phase

**Figure 8:**
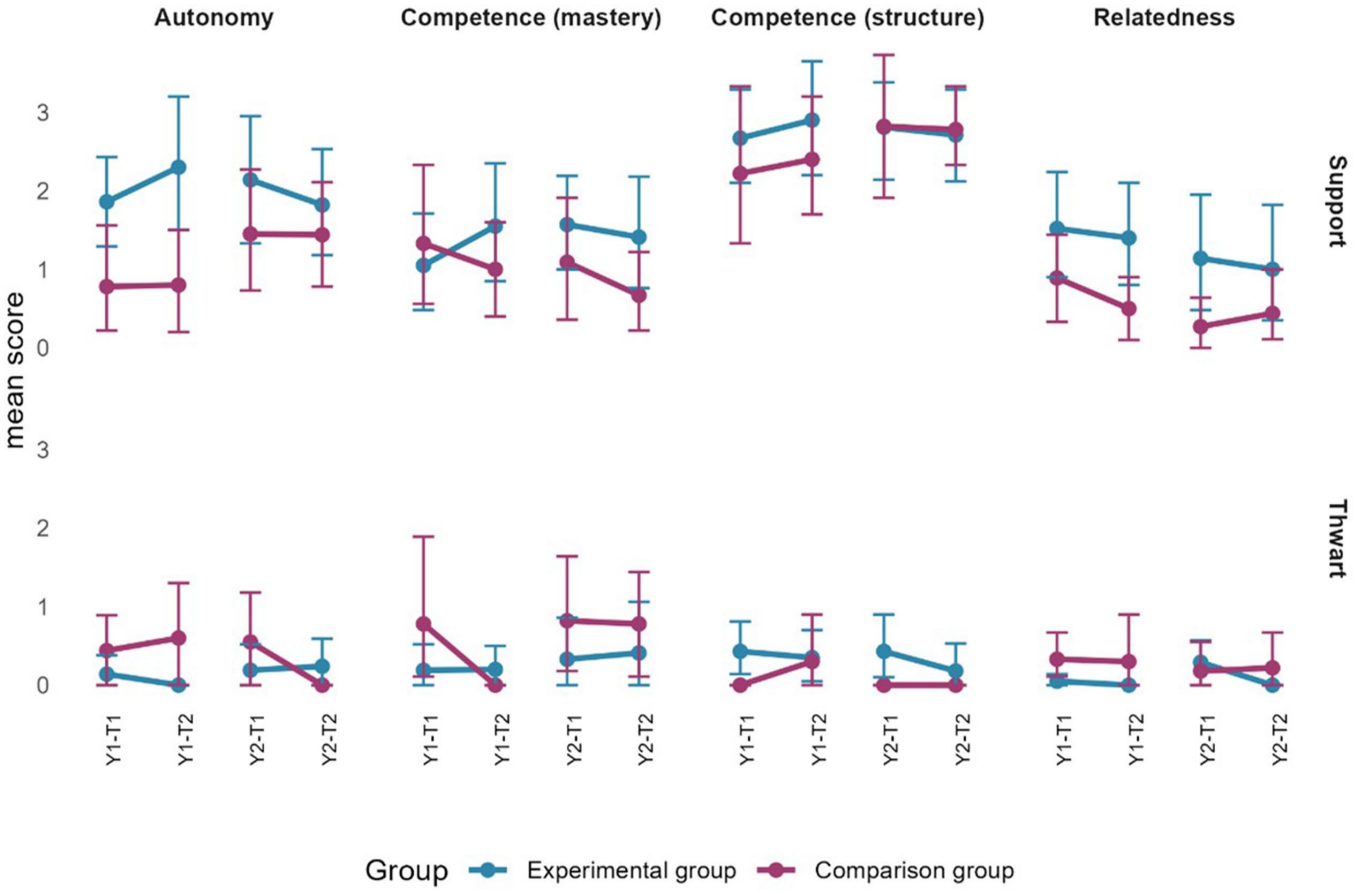
Evolution of observed motivational climate during the integration phase between the beginning and end of each year for each group. Error bars represent 95% confidence intervals estimated by bootstrap (*n* = 2000).

For teachers in the EG, the average duration of the integration phase varied between 1.12 and 5.97 min in year 1 and between 0 and 9.23 min in year 2. For teachers in the CG, in year 1, the average duration varied between 1.27 and 4.73 min in year 1 and, between 1.38 and 4.62 min in year 2. As Figure 8 shows, the integration phase is characterized by a high degree of similarity between the two groups for both need-thwarting and need-support. Indeed, all error bars overlap.

#### Total lesson

**Figure 9:**
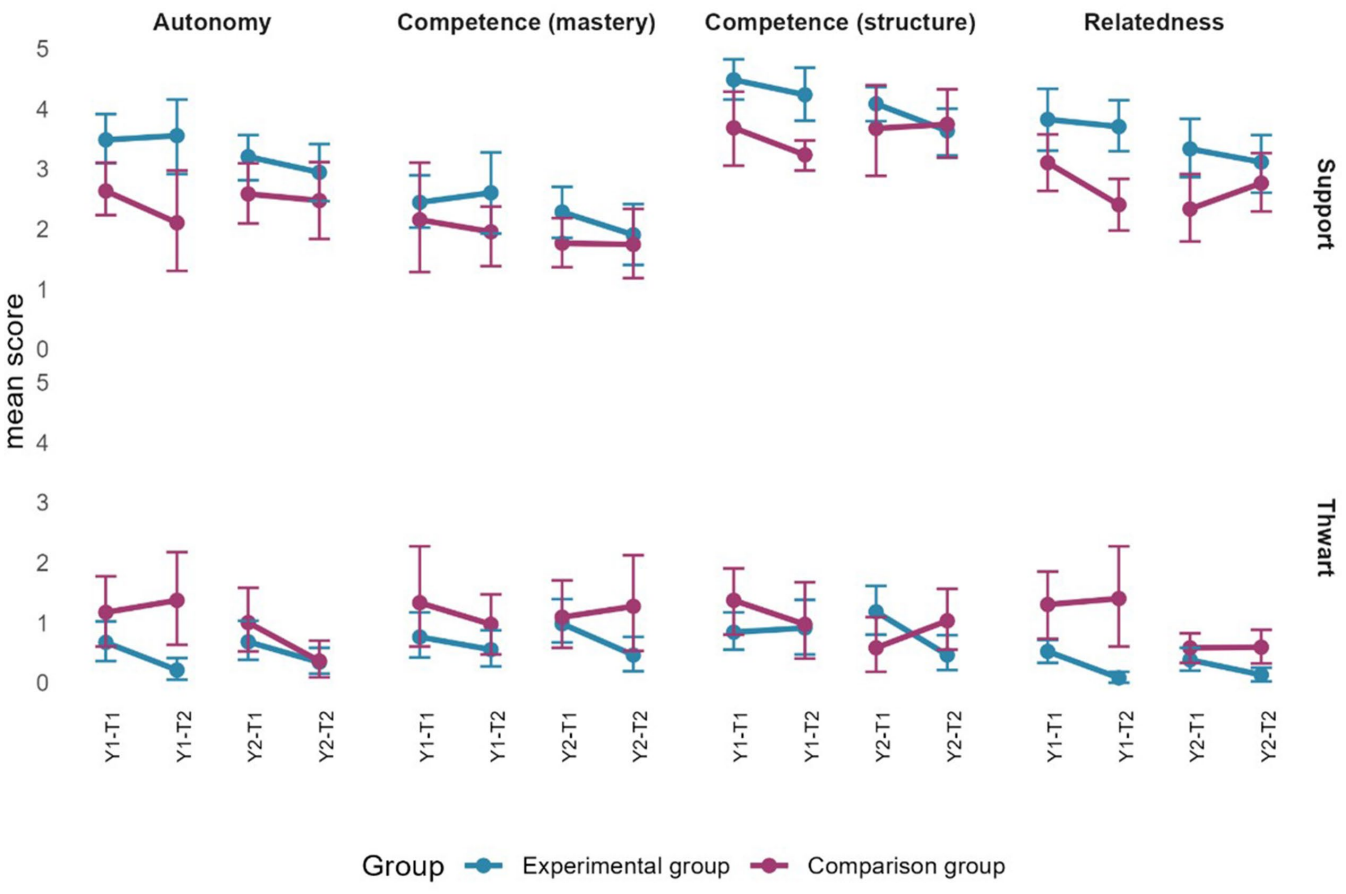
Evolution of observed motivational climate through time for the total lesson. Error bars represent 95% confidence intervals estimated by bootstrap (*n* = 2000).

Figure 9 presents the mean scores for the total lesson (excluding gaps). Overall, need-support from teachers in the EG appears higher than from those in the CG. However, confidence intervals are non-overlapping only at the end of the first year: autonomy- and relatedness-thwarting were higher from teachers in the CG, while teachers in the EG showed higher support for these needs. Moreover, competence-support through structure was higher from teachers in the EG at the end of the first year. Finally, relatedness thwarting was higher in the CG at the end of the second year.

#### Gaps

**Figure 10:**
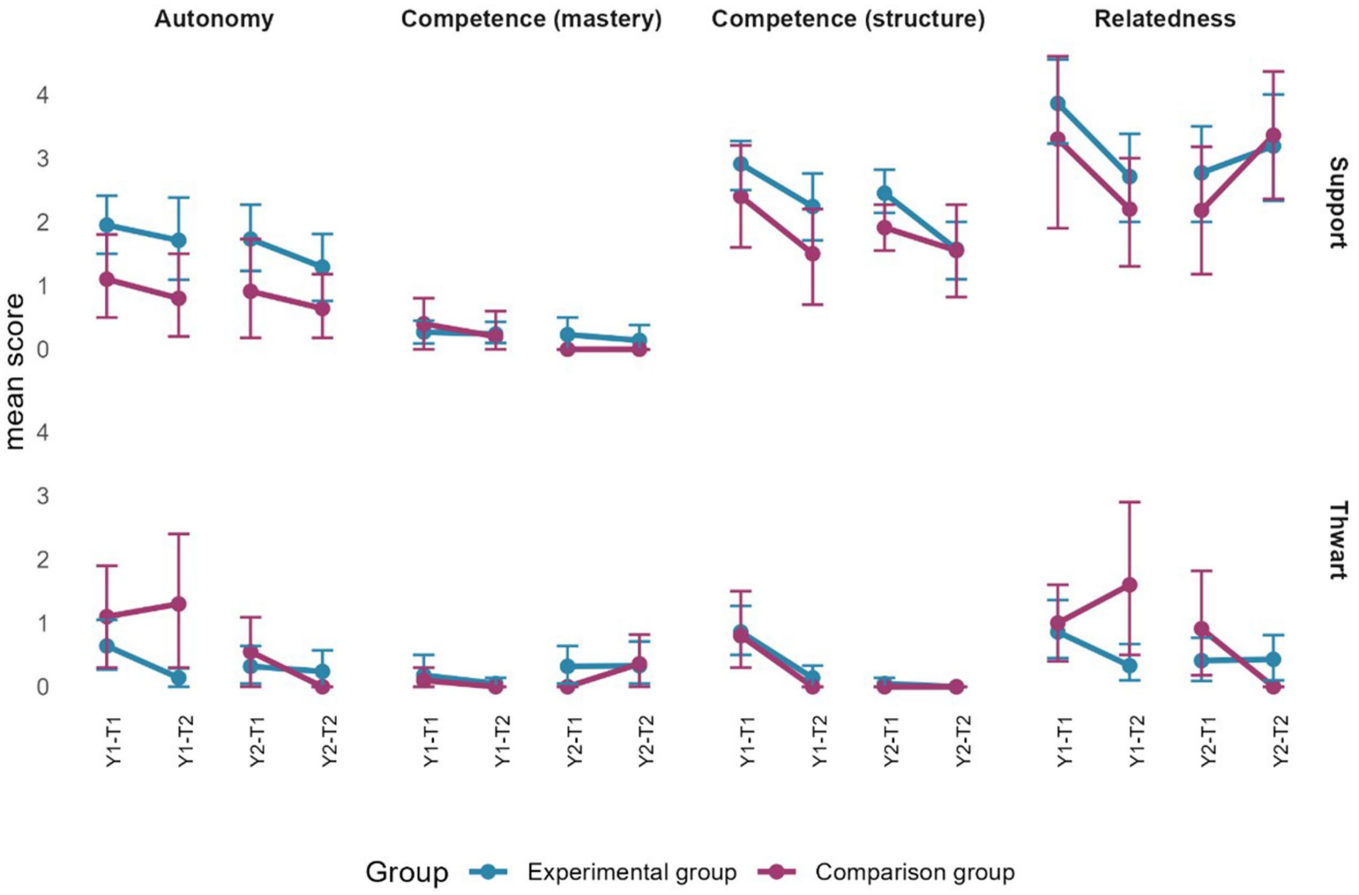
Evolution of observed motivational climate during the gaps between the beginning and end of each year for each group. Error bars represent 95% confidence intervals estimated by bootstrap (*n* = 2000).

For teachers in the EG, the average duration of gaps varied between 0.83 and 10.43 min in year 1 and between 3.62 and 14.40 min in year 2. For teachers in the CG, the average duration varied between 2.27 and 19.73 min in year 1, and between 3.37 and 13.27 min in year 2. According to Figure 10, gaps also highlight many similarities between the two groups. Only autonomy-thwarting is higher for teachers in the CG at the end of the first year.

#### Effect sizes for between-group comparisons

Table 6 presents a summary of effect sizes higher than Hattie’s (2012) threshold. Effect sizes should be negative for thwarting dimensions of the motivational climate and positive for support.

**Table 6:** Effect sizes for between-group comparisons.

Dimensions of the motivational climate	Cohen’s *d*	Phase	Autonomy	Competence (structure)	Competence (mastery)	Relatedness	Total
Support	> 0.4	Preparation	3	3	2	4	12
		Realization	1	2	0	1	4
		Integration	2	0	1	4	7
		Total lesson	4	3	2	3	12
		Gaps	4	3	1*	0	8
		Total	14	11	6	12	43
Thwart	> 0.4	Preparation	0	0	0	0	0
		Realization	0	1	0	0	1
		Integration	1*	2*	0	0	3
		Total lesson	0	1	0	0	1
		Gaps	1*	1*	1*	1*	4
		Total	2	5	1	1	9
	< −0.4	Preparation	2	2	2	2	8
		Realization	2	2	2	3(+1*)	10
		Integration	2(+1*)	0	2	1(+2*)	8
		Total lesson	2	2	3	4	11
		Gaps	2	0	0	2	4
		Total	11	6	9	15	41

*Effect sizes were calculated with one of the two groups having zero variance. They should be interpreted with caution due to undefined or inflated estimates.

Approximately half of the effect sizes support our hypotheses (54% for support, 51% for thwarting) with an effect size above the proposed threshold. For thwarting, approximately 11% correspond to effects contrary to what is expected. However, the vast majority (78%) were calculated from one of the two groups having 0 variance, making the effect size calculation less reliable.

## Discussion

Although the project was initially designed to evaluate the outcomes of follow-up support provided to PE teachers, the intervention could not be fully described in terms of dose or fidelity due to methodological contingencies and contextual variability. Accordingly, the findings should be interpreted as insights into implementation processes within a partnership-based, action-oriented research framework, rather than as evidence of efficacy. In this context, the differentiated follow-up support offered to some teachers created conditions that allowed us to examine whether those operating within a structured support model (see Table 4) differed from teachers not involved in the project. Thus, the observed changes cannot be systematically attributed to a specific, standardized intervention; rather, they should be interpreted in terms of differences between two contextual conditions.

### Pupils’ motivational perceptions

In response to objectives 1 and 2 regarding pupils’ outcomes, results agree with previous studies showing that pupils rarely notice the changes observed and reported by teachers (Weaver et al., 2018; Borghouts et al., 2021). A reason for this may be the challenge of collecting data on pupils’ perceptions. Although the short French version of the Motivational Climate Questionnaire-PE (SFMCQ-PE; Mastagli et al., 2026) was used for the first time to measure change in pupils’ perception of empowering and disempowering motivational climates, the results are consistent with those reported in studies using other questionnaires previously validated in English. These include the Empowering and Disempowering Motivational Climate Questionnaire for coaches (EDMCQ-C; Appleton et al., 2016), used in the study by Girard et al. (2023b), and the Empowering and Disempowering Motivational Climate Questionnaire for PE (EDMCQ-PE; Milton et al., 2018), used in Milton et al. (2025a). A French version of the EDMCQ-PE was validated (Simon et al., 2025a), and another questionnaire has also been developed in English (the MUMOC-PES; Vlachos and Papaioannou, 2023). However, to date, neither instrument has been used to evaluate intervention effects. As for theory-based professional development programs aimed at promoting a more empowering motivational climate in PE, whether training was provided by a researcher (Milton et al., 2025a) or collaboratively by researchers and ECs (Hogue, 2022; Girard et al., 2023b), findings consistently point to minimal observable changes among pupils (Girard et al., 2023b; Milton et al., 2025a), despite teachers’ reports of benefits from implementing motivational strategies and perceived improvements in pupils’ motivation and engagement (Hogue, 2022; Milton et al., 2025a).

Overall, many studies have attempted to evaluate pupils’ motivation and engagement toward PE using different types of data collection methods (e.g., questionnaires, individual interviews, focus groups, systematic observations) (Girard et al., 2023b; Milton et al., 2025a; Girard et al., 2025). Yet, the results of such studies never appear to reflect and actually capture what teachers report in terms of their experiences in the field (e.g., disengaged pupils) and what objective data indicate regarding pupils’ physical inactivity and sedentary levels (Aelterman et al., 2012, Valladares-Garrido et al., 2025; World Health Organization, 2022). In other words, even if teachers perceive their pupils as unmotivated and disengaged, or if systematic observations indicate that some teachers use practices that have the potential to thwart pupils’ basic psychological needs, this does not seem to be reflected in pupils’ responses. It is important to acknowledge that pupils’ self-reported perceptions of their motivation toward PE do not necessarily translate into corresponding behaviors within or outside of PE classes. To examine the potential discrepancies between perceived and actual motivation, a mixed-method approach could be employed. For instance, pupils could be asked to complete standardized questionnaires, be video-recorded during PE, and subsequently participate in stimulated-recall interviews aimed at exploring divergences between their self-perceptions and observed behaviors, thereby shedding light on the underlying mechanisms explaining such discrepancies. In addition, collecting teachers’ assessments of each pupil’s motivation and engagement through parallel questionnaires would allow for a systematic comparison of teacher- and pupil-reported perceptions, and more importantly, provide insights into the origins of these perceptual differences. All this underscores that taking pupils’ perceptions into consideration is complicated and requires in-depth and careful reflection on how such data should be collected. In sum, although student self-report questionnaires are appropriate for documenting overall perceptions, they may be less sensitive to detecting subtle, gradual, and contextually variable changes in teaching practices, particularly in an ecological setting. Accordingly, our research efforts should focus on PE teachers’ evidence-based practices and behaviors (i.e., empowering motivational strategies), with the aim of fostering a cultural shift in teaching practices within the field. In this regard, student data was complemented by other data collection methods, such as systematic observations.

### Observed implementation of the motivational climate

In line with the above and in response to objectives 3 and 4, few meaningful changes were observed in certain dimensions of the motivational climate established by teachers in the EG. This indicates they were more empowering and less disempowering, although most dimensions of the motivational climate implemented remained stable over time. In general, teachers in the EG tended to be more empowering and less disempowering throughout the 2 years of data collection. Indeed, some comparisons between groups at each measurement point indicated effects that were over the hinge point suggested by Hattie (2012). Specifically, he found that the average effect size of interventions in the educational context was 0.40, which, from his point of view, could be used as a comparison point to identify interventions that provided effects over or under that mean. In other words, reaching an effect over this hinge point could be a sign of “visible learning” by teachers (Hattie, 2012). Nevertheless, it should not be forgotten that this heuristic benchmark, does not provide clear evidence of learning attributable to the intervention, especially under conditions as variable as those experienced in the project.

Consistent with the above, the complexity of the realities faced by teachers, ECs, and researchers must be considered to explain our results, as these contextual factors directly influence what can realistically be observed and measured. First, we suggest that one reason for the modest changes over time may be that observations represent only a fixed point in time and so may not fully capture teachers’ positive attempts to implement empowering motivational strategies. Indeed, professional learning and, more specifically, in-service learning are dynamic processes shaped by trial and error and multiple cycles of experimentation (Desbiens et al., 2023). As such, video recordings may not have fully captured their iterative attempts and practice effort. This also means that teachers’ learning trajectories may unfold in ways that cannot be immediately observed in isolated recordings. In addition, during the development of the Multidimensional Motivational Climate Observation System (MMCOS; Sarrazin et al., 2015) designed to capture empowering and disempowering motivational climates in the sport context, the authors raised the question of the relevance of identifying which motivational strategy contributes most strongly to the rating of a given motivational climate dimension, in order to better inform and guide interventions. These considerations may prompt a re-examination of the way observations are coded or encourage efforts to reduce the number of motivational strategies observed per dimension by prioritizing those that appear most influential. In this regard, previous work has often suggested that disempowering strategies have a more immediate and negative impact on students, whereas empowering motivational strategies require a stable context on a longer time span to yield observable positive effects on students (Papaioannou et al., 2007; Cheon et al., 2012; Vansteenkiste and Ryan, 2013; Cheon et al., 2016). Similarly, to observe changes in teachers’ practices, and in line with results from longitudinal and individual case studies (Desimone et al., 2002; Kang, 2025), we believe that observation would benefit from methodological approaches that are better adapted to capturing gradual and individualized changes (e.g., longitudinal and case studies) (Short et al., 2017). Second, context must also be taken into consideration given that a major strike occurred during the first year of the study. For some teachers, this called for adjustments in the number of video recordings and the moment of recording. PE teachers were also forced to make important modifications to their planning, which may have affected the motivational climate established. These results are consistent with those of Girard et al. (2025), who demonstrate that pupils’ engagement is sometimes higher than usual during a crisis, as they regain interest in participating when opportunities that had previously been interrupted are reintroduced. Third, in each academic year of the project, teachers were asked to select a new group of pupils to participate in the study. Thus, they had to deal with different types of pupils’ motivation, engagement, behaviors, and needs, possibly leading them to rely on more habitual motivational strategies (Ødegård and Solberg, 2024). These results are highly instructive when combined with those of Desbiens et al. (2013) regarding PE teachers’ strong conviction about the relevance of favoring looping with their pupils, i.e., teaching the same group of pupils for more than one school year. From the teachers’ perspective, looping allows them to develop high-quality, lasting, and significantly more productive relationships with their pupils. These results also highlight that creating a motivational climate conducive to pupils’ motivation and engagement takes time. Fourth, not all ECs had the same resources to support their teachers with comparable methods. Since we firmly believe that follow-up support should be adapted to the reality of everyone involved, this is an important factor to consider when interpreting our results, aligning as it does with Milton’s qualitative findings: “The longevity and varied interaction methods of the PDP contributed to its sustainability. Teachers appreciated the ability to ‘dip in and out’ of the programme, with regular posts and reminders keeping them focused and encouraging innovative thinking” (Milton et al., 2025a, p. 10). Finally, in the development of professional competencies, when changes in practice are just beginning to emerge, the collection of qualitative data may be particularly salient. Specifically, conducting individual or group interviews, as has been done in other studies (Hogue, 2022; Milton et al., 2025a, 2025c), may provide richer insights into teachers’ perspectives, their emerging awareness, and the ways in which this awareness is manifested in their daily professional practices, rather than being captured solely during moments of systematic observation. It is also possible that the magnitude of the changes required for some teachers, and possibly for some ECs, was underestimated at the outset of the project. This highlights the need for a systematic assessment of training needs early in the process, for both teachers and ECs, to provide support of sufficient intensity. For instance, study outcomes might have differed had ECs been required, from the first year of follow-up support, to conduct in-gymnasium observations and provide individualized feedback. Such an approach would also have required stronger initial support from the research team.

Changing teaching practices is clearly a long and complex process (Morina et al., 2025). According to Guay and Prud'homme (2018), a minimum of 3 years is often necessary to observe significant changes. Additionally, because changing teaching practices requires time and support from professionals (Daigle et al., 2021; Kraft et al., 2018), such as ECs. Therefore, more quality time must be allowed for mentorship (Makopoulou et al., 2019). Our results suggest that although ECs were expected to support teachers in transferring theory into practice, they became aware they had not fully integrated certain theoretical concepts and, in this regard, were likewise in need of support, as it was previously reported by ECs from Ontario (Canada) (Duchesne, 2016). Here a critical point must be made: although ECs are tasked with advising, training, supporting, and innovating (Guillemette et al., 2019), they may struggle to fulfil these roles effectively in terms of motivational theoretical concepts. Thus, offering sustained support is essential, not only to strengthen their own understanding, but also to enhance their capacity to help teachers foster a more empowering and less disempowering motivational climate. These results are consistent with previous work showing that ECs sometimes struggle to fully assume their role as content and field specialists, notably because “they do not always possess the in-depth knowledge or experience that is attributed to them” (Duchesne, 2016, p. 647). This issue is particularly salient given that perceived “coach” expertise (both from teachers’ and ECs’ perspective) shape effectiveness of “coaching” support (Smith and Desimone, 2025). Furthermore, ECs express concerns about how to provide feedback and establish their credibility (Duchesne, 2016), as well as a need for additional preparation to better train and support adult learners (Lachaîne and Duchesne, 2020). In line with these observations, the present study contributes to a better understanding of the benefits associated with investing ECs’ engagement in research activities, a dimension of their role that remains underutilized (Duchesne, 2016), and which may enhance their ability to enact their complementary responsibilities (Guillemette et al., 2019).

Taken together, these results highlight the need for a cultural shift in both pre-service and in-service teacher training if pupils are to experience meaningful changes in the motivational climate. Pre-service teacher training in Quebec reflects a culture that encourages practices conducive to an empowering motivational climate. This orientation was further strengthened by the introduction of the revised professional competency framework for the province’s teaching profession, which now includes a specific competency aimed at fostering student motivation – namely, *support pupils’ love of learning* (Ministère De L'éducation, 2020). Aligning pre-service and in-service training is therefore essential to promote a genuine cultural shift. In this regard, evaluation of the recent initiative *Learning how to motivate* offers promising results in terms of supporting pre-service PE teachers in implementing an empowering motivational climate during their final secondary school internships (Girard and de Guise, 2024) and impacting their beliefs about empowering and disempowering motivational strategies (Girard et al., 2026).

### Limitations and strengths of the study

To conduct research in schools while respecting teachers’ complex reality necessarily involves limitations as regards methodology. Thus, self-reported measures such as questionnaires can be affected by a social desirability bias, which may explain the high scores obtained on variables favorable to pupils’ motivation and engagement, leaving limited room for improvement. Moreover, because of the school-based nature of the research project, there is a selection bias in the composition of the sample. Indeed, teachers were recruited on a voluntary basis in a post-pandemic context. The small sample size of teachers from various background and with different characteristics (e.g., elementary vs. high school; inexperienced vs. experienced teachers) did not allow to verify if results vary according to these characteristics. In future studies, using larger samples could allow to test these hypotheses. Additionally, because teachers were not randomly distributed between the two groups (EG and CG), there were potential differences between participants from the outset of the project, which might result in a selection bias. Indeed, PE teachers in the EG were more likely to seek for a change in their pedagogical practices, and willing to invest time and energy in that matter. Furthermore, the composition of the groups of teachers (and pupils) changed between the 2 years, meaning that the planned two-year longitudinal follow-up had to be considered as two 1-year follow-ups instead. On one hand, all these aspects represent design limitations and results should therefore be interpreted with caution. On the other hand, they also highlight one of the strengths of the project: it allowed us to maintain a strong ecological value while respecting the lived reality of teachers and ECs. This also led to another potential limitation: although the statistical analyses conducted highlight certain salient differences, further research is needed to draw more precise conclusions given the many changes that occurred during the project and the limited number of teachers involved. From a research perspective, testing specific intervention methods in a standardized and controlled context should be considered. Indeed, a critical consideration concerns the delivery of the *Motivate to learn* training during the provincial rollout of the training (2022–2023). Specifically, the condensed online training delivered province-wide by multiple ECs, without ongoing support from the research team, may have been implemented with lower fidelity than the original pilot training (full in-person version). This likely contributed to an initial emphasis on content reappropriation rather than sustained implementation in practice during the first year of follow-up support of the present project. Moreover, the scope and intensity of the support may not have sufficiently accounted for the magnitude of change required for some teachers and ECs, underscoring the importance of conducting a thorough assessment of training needs early in the intervention design. However, in applied research, it is equally essential to conduct studies in partnership with the communities involved to enhance the ecological validity and increase its practical relevance and acceptability by participants. Indeed, by analyzing up to 130 videos, the study generated an impressive amount of data, and the heterogeneity of PE teachers’ support modalities and individualized objectives were highly enriching and compatible with their realities. Nevertheless, this greatly limited the possibility of evaluating the changes statistically.

To conclude, the present study was not conducted under favorable conditions and encountered many challenges, a situation generally representative of action research in real life school-based settings. Nevertheless, this two-year project proved a highly enriching experience for both practitioners and researchers. Both parties recognized the need to continue efforts over a longer period in view of the uncertainties inherent in working to support the development of teachers’ professional competencies. Finally, an innovative and unexpected insight obtained was ECs’ urgent need for support from the research team in providing individualized assistance to teachers implementing an empowering motivational climate. Given that the working conditions and needs of ECs in PE is a new field of research in Quebec, our results show that further attention from researchers is required. For instance, future research could focus on developing ECs’ competent action, as conceptualized by Guillemette et al. (2019). Toward this end, the present study underscores the necessity of working in collaboration with ECs to assist them in appropriating theoretical content and developing teacher support (e.g., observations, questioning, feedback).

## Data Availability

The datasets presented in this article are not readily available because they are stored by the research team in accordance with the standards set out in the ethical consent form. Requests to access the datasets should be directed to stephanie.girard3@uqtr.ca.

## Footnotes

1 Direction des programmes d’enseignement en formation générale des jeunes and Direction sport, loisir, activité physique et plein air.
