# Supplementary material for: Supporting physical education teachers to create an empowering motivational climate
Source: Front Psychol. 2026 May 15;17:1771885. doi: 10.3389/fpsyg.2026.1771885 (PMC13218922; doi:10.3389/fpsyg.2026.1771885)
Supplement: Supplementary file 1 [file Supplementary_file_1.pdf]

Supplementary file A. Observation grid (adapted from Masked for review)

Two strategies were adapted from the original grid in the Competence support (mastery; strategy 1) and in the Competence support (structure; strategy 4) dimensions.

**Teacher observed:**			
**Empowering motivational climate score:**			
**Preparation / Realization / Integration / Gaps**			
0 = not at all<br>*1 = very low*	*2 = low*<br>*3 = moderately low*	*4 = moderate*<br>*5 = moderately strong*	*6 = strong*<br>*7 = very strong*
**Autonomy support**: 0 1 2 3 4 5 6 7			
1. Acknowledges pupils' interests, feelings and perspective.<br>*2. Provides rationale for requests and constraints.*<br>*3. Explains tasks and exercises' importance, utility and significance.*<br>*4. Provides meaningful choice to pupils.*<br>*5. Gives pupils the opportunity to practice independently and to solve problems on their own, without interfering.*<br>*6. Encourages initiative taking.*<br>7. *Provides opportunity for pupils input (e.g. give their opinion, make changes to tasks, make suggestions, etc.).*			
**Competence support (mastery)**: 0 1 2 3 4 5 6 7			
1. Uses different pupils as positive "role model".<br>*2. Provides tasks adapted to the multiple abilities of the pupils.*<br>*3. Provides variation between or within exercises.*<br>*4. Emphasizes task-focused positive competence feedback.*<br>*5. Emphasizes/recognizes effort and/or improvement.*<br>*6. Uses cooperative learning.*<br>*7. Applies differentiation.*<br>8. *Emphasizes effort and engagement in the learning process rather than student performance.*			
**Competence support (structure)**: 0 1 2 3 4 5 6 7			
1. Gives an overview of the content and structure of the lesson.<br>*2. Gives clear instructions.*<br>*3. Offers expectations for learning.*<br>*4. Use demonstration to help pupils understand what they need to do.*<br>*5. Monitors if pupils consequently live up to the instructions.*<br>*6. Offers help and gives tips and advice during activities.*<br>*7. Reviews with pupils the overall lesson content and structure.*<br>8. *Questions pupils on what they have learned during the lesson.*			
**Relatedness support**: 0 1 2 3 4 5 6 7			

1. Ensures all pupils are included and respected in the group.<br>*2. Is enthusiastic and eager.*<br>*3. Puts effort and energy into the facilitation and conduct of the lesson.*<br>*4. Adopts a warm communication style.*<br>*5. Engages in noninstructional conversation with pupils.*<br>*6. Pays attention to what pupils are saying.*<br>*7. Shows care and concern for pupils.*<br>*8. Addresses pupils by their first name when the opportunity occurs.*<br>*9. Is empathic.*<br>*10. Is physically and psychologically close to pupils.*<br>*11. Shows unconditional regard towards all pupils.*	

**Disempowering motivational climate score:**			
**Preparation / Realization / Integration / Gaps**			
0 = not at all<br>*1 = very low*	*2 = low*<br>*3 = moderately low*	*4 = moderate*<br>*5 = moderately strong*	*6 = strong*<br>*7 = very strong*
**Control**: 0 1 2 3 4 5 6 7			
1. Uses controlling strategies (e.g. make all decisions, threat to punish, etc.)<br>*2. Uses extrinsic rewards (e.g. promises, rewards, consequences, etc.).*<br>*3. Relies on authority in response to pupils' complaints/requests.*			
**Performance**: 0 1 2 3 4 5 6 7			
1. Emphasizes/recognizes inferior/superior performance and ability.<br>*2. Encourages rivalry between pupils.*<br>3. *Emphasizes errors and/or performance.*			
**Chaos**: 0 1 2 3 4 5 6 7			
1. Gives few or no explanations or they are imprecise.<br>*2. Leaves pupils to themselves during the task.*<br>3. *Demonstrates little consistency and coherence/is unpredictable.*			
**Relatedness Thwarting**: 0 1 2 3 4 5 6 7			
1. Uses strategies/activities allowing the exclusion of certain pupils.<br>*2. Restricts opportunities for interactions and conversation "with" and "between" pupils.*<br>*3. Is distant from pupils.*<br>4. *Uses sarcasm.*			

Example of coding 1:

A teacher scored 3/7 in competence need thwarting (performance climate) during the realization phase. Of the three tasks proposed, one mainly benefited higher-performing students, and feedback was largely outcome-oriented, with praise directed primarily at those who were able to stay in the game. However, in the two other tasks, the teacher did not adopt such practices (which explains the moderate score assigned for competence need thwarting), used several students as positive models, and emphasized students' efforts. These practices resulted in a score of 4/7 for competence support.

Example of coding 2:

A teacher scored 7/7 in autonomy support during the preparation phase. He consistently explained the reasons behind imposed requirements and clarified the relevance of the proposed tasks in terms of how they would help students mobilize their learning. He offered meaningful choices within a clearly structured task, used questioning to prompt students to identify answers by themselves and stimulate reflection, and allowed them to adjust elements of the task based on their suggestions, thereby fostering initiative. At no point did he rely on controlling strategies or external motivational pressures, which resulted in a score of 0/7 for autonomy need thwarting.
