# Supplementary material for: Supporting physical education teachers to create an empowering motivational climate
Source: Front Psychol. 2026 May 15;17:1771885. doi: 10.3389/fpsyg.2026.1771885 (PMC13218922; doi:10.3389/fpsyg.2026.1771885)
Supplement: Supplementary file 2 [file Supplementary_file_2.pdf]

**Supplementary file B. Tables presenting effect sizes for between groups comparison**

Table B1. Effect sizes for the evolution of pupils' perceptions

| Year | Motivational variables | EG<br>*M*(*sd*) | CG<br>*M*(*sd*) | Cohen's d |
|---|---|---|---|---|
| 1 | Empowering climate | 0.00 (1.08) | -0.09 (1.24) | 0.08 |
| | Disempowering climate | 0.05 (1.12) | 0.34 (1.32) | -0.24 |
| | Autonomy | 0.22 (1.22) | 0.15 (1.54) | 0.05 |
| | Competence | -0.03 (1.22) | -0.08 (1.22) | 0.04 |
| | Relatedness | 0.07 (1.37) | 0.20 (1.34) | -0.10 |
| | Mastery goals | -0.02 (1.38) | 0.03 (1.16) | -0.04 |
| | Performance-approach goals | 0.26 (1.83) | 0.39 (1.95) | -0.07 |
| | Performance-avoidance goals | 0.12 (1.96) | 0.30 (1.94) | -0.09 |
| | Self-determined motivation | 0.04 (1.20) | 0.18 (1.18) | -0.12 |
| | Controlled motivation | -0.09 (1.14) | 0.01 (1.20) | -0.08 |
| | Amotivation | 0.04 (1.58) | 0.17 (1.75) | -0.08 |
| | Effort | -0.11 (1.29) | 0.19 (1.14) | -0.24 |
| | Intention | 0.23 (1.15) | 0.09 (0.88) | 0.13 |
| 2 | Empowering climate | -0.25 (1.36) | -0.06 (1.23) | -0.14 |
| | Disempowering climate | -0.05 (1.69) | 0.10 (1.23) | -0.09 |
| | Autonomy | 0.02 (1.88) | -0.06 (1.28) | 0.05 |
| | Competence | -0.18 (1.46) | -0.15 (1.23) | -0.02 |
| | Relatedness | -0.03 (1.56) | -0.13 (1.29) | 0.07 |
| | Mastery goals | -0.28 (1.39) | 0.03 (1.21) | -0.23 |
| | Performance-approach goals | 0.01 (1.71) | 0.14 (1.63) | -0.07 |
| | Performance-avoidance goals | -0.30 (1.85) | -0.31 (1.68) | 0.00 |
| | Self-determined motivation | -0.21 (1.23) | 0.09 (0.90) | -0.27 |
| | Controlled motivation | -0.22 (1.26) | -0.08 (1.34) | -0.11 |
| | Amotivation | 0.05 (1.61) | -0.36 (1.55) | 0.26 |
| | Effort | -0.15 (1.18) | 0.04 (1.37) | -0.16 |
| | Intention | 0.16 (1.40) | -0.05 (1.17) | 0.15 |

**Differences in teacher's instauration of empowering climate**

Table B2. Effect sizes for the preparation phase

| Year | Time of measurement | | Dimension | EG M(sd) | CG M(sd) | Cohen's d |
|---|---|---|---|---|---|---|
| 1 | 1 | Support | Autonomy | 4.59 (1.18) | 3.22 (1.39) | 1.10 |
| | | | Competence (mastery) | 2.23 (1.31) | 1.44 (1.42) | 0.58 |
| | | | Competence (structure) | 5.55 (0.74) | 4.56 (1.42) | 1.01 |
| | | | Relatedness | 4.32 (1.52) | 2.89 (1.36) | 0.96 |
| | | Thwart | Autonomy | 0.73 (1.16) | 1.00 (1.00) | -0.24 |
| | | | Competence (mastery) | 0.50 (1.06) | 1.00 (1.32) | -0.44 |
| | | | Competence (structure) | 0.82 (1.22) | 1.67 (1.41) | -0.66 |
| | | | Relatedness | 0.73 (0.83) | 1.11 (1.05) | -0.43 |
| | 2 | Support | Autonomy | 3.82 (1.97) | 3.20 (1.55) | 0.33 |
| | | | Competence (mastery) | 2.41 (1.79) | 1.80 (1.55) | 0.35 |
| | | | Competence (structure) | 5.23 (1.41) | 4.30 (0.67) | 0.75 |
| | | | Relatedness | 4.27 (1.49) | 3.00 (1.15) | 0.91 |
| | | Thwart | Autonomy | 0.32 (0.84) | 1.50 (1.72) | -1.01 |
| | | | Competence (mastery) | 0.45 (0.96) | 1.40 (1.51) | -0.82 |
| | | | Competence (structure) | 1.09 (1.38) | 1.40 (1.65) | -0.21 |
| | | | Relatedness | 0.09 (0.29) | 2.00 (1.89) | -1.80 |
| 2 | 1 | Support | Autonomy | 3.91 (1.48) | 2.45 (1.44) | 0.99 |
| | | | Competence (mastery) | 2.09 (1.34) | 1.45 (1.21) | 0.49 |
| | | | Competence (structure) | 5.50 (0.91) | 4.27 (1.56) | 1.06 |
| | | | Relatedness | 4.23 (1.34) | 2.45 (1.44) | 1.29 |
| | | Thwart | Autonomy | 0.86 (1.13) | 0.91 (1.22) | -0.04 |
| | | | Competence (mastery) | 1.00 (1.27) | 0.64 (1.12) | 0.30 |
| | | | Competence (structure) | 1.23 (1.72) | 0.64 (1.43) | 0.36 |
| | | | Relatedness | 0.41 (0.80) | 0.64 (1.21) | -0.24 |
| | 2 | Support | Autonomy | 3.62 (1.75) | 2.36 (1.29) | 0.78 |
| | | | Competence (mastery) | 1.71 (1.79) | 1.55 (1.04) | 0.11 |
| | | | Competence (structure) | 4.29 (1.59) | 4.64 (1.03) | -0.25 |
| | | | Relatedness | 3.38 (1.75) | 2.45 (1.75) | 0.53 |
| | | Thwart | Autonomy | 0.14 (0.36) | 0.64 (1.03) | -0.75 |
| | | | Competence (mastery) | 0.52 (0.93) | 0.91 (1.22) | -0.37 |
| | | | Competence (structure) | 0.43 (1.08) | 1.00 (1.41) | -0.48 |
| | | | Relatedness | 0.33 (0.73) | 0.36 (0.50) | -0.05 |

Table B3. Effect sizes for the realization phase

| Year | Time of measurement | | Dimension | EG<br>*M*(*sd*) | CG<br>*M*(*sd*) | Cohen's d |
|---|---|---|---|---|---|---|
| 1 | 1 | Support | Autonomy | 3.82 (1.65) | 3.70 (1.49) | 0.07 |
| | | | Competence (mastery) | 3.91 (1.74) | 3.40 (2.32) | 0.26 |
| | | | Competence (structure) | 5.09 (1.11) | 4.10 (1.97) | 0.70 |
| | | | Relatedness | 5.36 (1.40) | 5.30 (1.95) | 0.04 |
| | | Thwart | Autonomy | 1.14 (1.36) | 2.00 (1.94) | -0.56 |
| | | | Competence (mastery) | 1.59 (1.62) | 1.90 (1.91) | -0.18 |
| | | | Competence (structure) | 1.27 (1.16) | 2.40 (1.65) | -0.85 |
| | | | Relatedness | 0.77 (0.92) | 2.30 (2.00) | -1.14 |
| | 2 | Support | Autonomy | 4.32 (1.86) | 2.30 (2.21) | 1.02 |
| | | | Competence (mastery) | 3.59 (2.02) | 3.22 (2.05) | 0.18 |
| | | | Competence (structure) | 4.41 (1.47) | 3.00 (1.15) | 1.02 |
| | | | Relatedness | 5.14 (0.94) | 3.70 (1.49) | 1.26 |
| | | Thwart | Autonomy | 0.27 (0.77) | 2.00 (2.00) | -1.36 |
| | | | Competence (mastery) | 0.91 (1.27) | 1.50 (1.72) | -0.42 |
| | | | Competence (structure) | 1.18 (1.65) | 1.20 (1.48) | -0.01 |
| | | | Relatedness | 0.09 (0.29) | 1.90 (2.18) | -1.48 |
| 2 | 1 | Support | Autonomy | 3.64 (1.65) | 3.82 (1.83) | -0.11 |
| | | | Competence (mastery) | 3.18 (1.30) | 2.73 (1.56) | 0.33 |
| | | | Competence (structure) | 3.86 (1.17) | 3.91 (1.97) | -0.03 |
| | | | Relatedness | 4.50 (1.60) | 4.27 (1.79) | 0.14 |
| | | Thwart | Autonomy | 1.00 (1.31) | 1.55 (1.63) | -0.38 |
| | | | Competence (mastery) | 1.59 (1.30) | 1.82 (1.66) | -0.16 |
| | | | Competence (structure) | 1.86 (1.81) | 1.09 (1.58) | 0.44 |
| | | | Relatedness | 0.41 (0.73) | 0.91 (0.94) | -0.62 |
| | 2 | Support | Autonomy | 3.35 (1.98) | 3.27 (1.90) | 0.04 |
| | | | Competence (mastery) | 2.60 (1.98) | 2.64 (1.50) | -0.02 |
| | | | Competence (structure) | 3.90 (1.29) | 3.55 (1.51) | 0.26 |
| | | | Relatedness | 4.45 (1.73) | 4.73 (1.90) | -0.15 |
| | | Thwart | Autonomy | 0.70 (1.13) | 0.45 (1.04) | 0.22 |
| | | | Competence (mastery) | 0.55 (1.00) | 1.91 (2.12) | -0.92 |
| | | | Competence (structure) | 0.85 (1.27) | 1.73 (1.90) | -0.58 |
| | | | Relatedness | 0.00 (0.00) | 1.09 (1.14) | -1.64 |

Table B4. Effect sizes for the integration phase

| Year | Time of measurement | | Dimension | EG *M*(*sd*) | CG *M*(*sd*) | Cohen's d |
|---|---|---|---|---|---|---|
| 1 | 1 | Support | Autonomy | 1.86 (1.35) | 0.78 (1.09) | 0.84 |
| | | | Competence (mastery) | 1.05 (1.53) | 1.33 (1.41) | -0.19 |
| | | | Competence (structure) | 2.67 (1.39) | 2.22 (1.64) | 0.30 |
| | | | Relatedness | 1.52 (1.60) | 0.89 (0.93) | 0.44 |
| | | Thwart | Autonomy | 0.14 (0.48) | 0.44 (0.73) | -0.54 |
| | | | Competence (mastery) | 0.19 (0.68) | 0.78 (1.64) | -0.56 |
| | | | Competence (structure) | 0.43 (0.81) | 0.00 (0.00) | 0.63 |
| | | | Relatedness | 0.05 (0.22) | 0.33 (0.50) | -0.88 |
| | 2 | Support | Autonomy | 2.30 (2.03) | 0.80 (1.14) | 0.84 |
| | | | Competence (mastery) | 1.55 (1.73) | 1.00 (1.05) | 0.36 |
| | | | Competence (structure) | 2.90 (1.71) | 2.40 (1.26) | 0.32 |
| | | | Relatedness | 1.40 (1.54) | 0.50 (0.71) | 0.68 |
| | | Thwart | Autonomy | 0.00 (0.00) | 0.60 (1.07) | -0.98 |
| | | | Competence (mastery) | 0.20 (0.62) | 0.00 (0.00) | 0.39 |
| | | | Competence (structure) | 0.35 (0.75) | 0.30 (0.95) | 0.06 |
| | | | Relatedness | 0.00 (0.00) | 0.30 (0.95) | -0.56 |
| 2 | 1 | Support | Autonomy | 2.14 (1.88) | 1.45 (1.37) | 0.40 |
| | | | Competence (mastery) | 1.57 (1.43) | 1.09 (1.38) | 0.34 |
| | | | Competence (structure) | 2.81 (1.50) | 2.82 (1.60) | -0.01 |
| | | | Relatedness | 1.14 (1.80) | 0.27 (0.65) | 0.57 |
| | | Thwart | Autonomy | 0.19 (0.68) | 0.55 (1.04) | -0.44 |
| | | | Competence (mastery) | 0.33 (1.11) | 0.82 (1.33) | -0.41 |
| | | | Competence (structure) | 0.43 (0.98) | 0.00 (0.00) | 0.54 |
| | | | Relatedness | 0.29 (0.72) | 0.18 (0.60) | 0.15 |
| | 2 | Support | Autonomy | 1.82 (1.47) | 1.44 (1.13) | 0.28 |
| | | | Competence (mastery) | 1.41 (1.50) | 0.67 (0.87) | 0.56 |
| | | | Competence (structure) | 2.71 (1.26) | 2.78 (0.83) | -0.06 |
| | | | Relatedness | 1.00 (1.58) | 0.44 (0.73) | 0.41 |
| | | Thwart | Autonomy | 0.24 (0.66) | 0.00 (0.00) | 0.43 |
| | | | Competence (mastery) | 0.41 (1.18) | 0.78 (1.09) | -0.32 |
| | | | Competence (structure) | 0.18 (0.73) | 0.00 (0.00) | 0.30 |
| | | | Relatedness | 0.00 (0.00) | 0.22 (0.67) | -0.58 |

Table B5. Effect sizes for the total lesson

| Year | Time of measurement | | Dimension | EG *M*(*sd*) | CG *M*(*sd*) | Cohen's d |
|---|---|---|---|---|---|---|
| 1 | 1 | Support | Autonomy | 3.48 (0.99) | 2.63 (0.73) | 0.93 |
| | | | Competence (mastery) | 2.44 (1.07) | 2.15 (1.53) | 0.24 |
| | | | Competence (structure) | 4.48 (0.83) | 3.68 (1.06) | 0.89 |
| | | | Relatedness | 3.82 (1.30) | 3.10 (0.79) | 0.61 |
| | | Thwart | Autonomy | 0.67 (0.81) | 1.17 (1.00) | -0.57 |
| | | | Competence (mastery) | 0.76 (0.93) | 1.33 (1.53) | -0.50 |
| | | | Competence (structure) | 0.84 (0.78) | 1.37 (0.91) | -0.64 |
| | | | Relatedness | 0.52 (0.47) | 1.30 (0.95) | -1.21 |
| | 2 | Support | Autonomy | 3.55 (1.52) | 2.10 (1.40) | 0.98 |
| | | | Competence (mastery) | 2.60 (1.64) | 1.95 (0.82) | 0.45 |
| | | | Competence (structure) | 4.23 (1.12) | 3.23 (0.45) | 1.04 |
| | | | Relatedness | 3.70 (1.04) | 2.40 (0.73) | 1.36 |
| | | Thwart | Autonomy | 0.21 (0.45) | 1.37 (1.32) | -1.41 |
| | | | Competence (mastery) | 0.55 (0.74) | 0.97 (0.88) | -0.53 |
| | | | Competence (structure) | 0.91 (1.11) | 0.97 (1.06) | -0.05 |
| | | | Relatedness | 0.08 (0.23) | 1.40 (1.53) | -1.54 |
| 2 | 1 | Support | Autonomy | 3.20 (0.95) | 2.58 (0.87) | 0.68 |
| | | | Competence (mastery) | 2.28 (1.03) | 1.76 (0.73) | 0.55 |
| | | | Competence (structure) | 4.08 (0.72) | 3.67 (1.34) | 0.42 |
| | | | Relatedness | 3.33 (1.21) | 2.33 (0.95) | 0.88 |
| | | Thwart | Autonomy | 0.68 (0.79) | 1.00 (0.98) | -0.37 |
| | | | Competence (mastery) | 0.98 (0.88) | 1.09 (1.00) | -0.12 |
| | | | Competence (structure) | 1.18 (0.99) | 0.58 (0.83) | 0.65 |
| | | | Relatedness | 0.38 (0.49) | 0.58 (0.45) | -0.42 |
| | 2 | Support | Autonomy | 2.94 (1.15) | 2.47 (1.15) | 0.41 |
| | | | Competence (mastery) | 1.90 (1.19) | 1.74 (1.04) | 0.14 |
| | | | Competence (structure) | 3.63 (0.98) | 3.74 (1.01) | -0.12 |
| | | | Relatedness | 3.11 (1.14) | 2.76 (0.89) | 0.33 |
| | | Thwart | Autonomy | 0.34 (0.52) | 0.36 (0.59) | -0.04 |
| | | | Competence (mastery) | 0.46 (0.65) | 1.27 (1.42) | -0.83 |
| | | | Competence (structure) | 0.46 (0.73) | 1.03 (0.89) | -0.73 |
| | | | Relatedness | 0.13 (0.29) | 0.59 (0.51) | -1.23 |

Table B6. Effect sizes for the preparation phase

| Year | Time of measurement | | Dimension | EG M(sd) | CG M(sd) | Cohen's d |
|---|---|---|---|---|---|---|
| 1 | 1 | Support | Autonomy | 1.95 (1.17) | 1.10 (1.10) | 0.74 |
| | | | Competence (mastery) | 0.27 (0.46) | 0.40 (0.70) | -0.24 |
| | | | Competence (structure) | 2.91 (1.02) | 2.40 (1.43) | 0.44 |
| | | | Relatedness | 3.86 (1.61) | 3.30 (2.31) | 0.30 |
| | | Thwart | Autonomy | 0.64 (0.95) | 1.10 (1.37) | -0.42 |
| | | | Competence (mastery) | 0.18 (0.66) | 0.10 (0.32) | 0.14 |
| | | | Competence (structure) | 0.86 (0.94) | 0.80 (1.03) | 0.07 |
| | | | Relatedness | 0.86 (1.13) | 1.00 (1.05) | -0.12 |
| | 2 | Support | Autonomy | 1.71 (1.52) | 0.80 (1.14) | 0.65 |
| | | | Competence (mastery) | 0.24 (0.44) | 0.20 (0.63) | 0.08 |
| | | | Competence (structure) | 2.24 (1.30) | 1.50 (1.27) | 0.57 |
| | | | Relatedness | 2.71 (1.65) | 2.20 (1.48) | 0.32 |
| | | Thwart | Autonomy | 0.14 (0.36) | 1.30 (1.77) | -1.13 |
| | | | Competence (mastery) | 0.05 (0.22) | 0.00 (0.00) | 0.26 |
| | | | Competence (structure) | 0.14 (0.36) | 0.00 (0.00) | 0.48 |
| | | | Relatedness | 0.33 (0.73) | 1.60 (2.07) | -0.97 |
| 2 | 1 | Support | Autonomy | 1.73 (1.32) | 0.91 (1.30) | 0.62 |
| | | | Competence (mastery) | 0.23 (0.61) | 0.00 (0.00) | 0.45 |
| | | | Competence (structure) | 2.45 (0.80) | 1.91 (0.70) | 0.71 |
| | | | Relatedness | 2.77 (1.85) | 2.18 (1.83) | 0.32 |
| | | Thwart | Autonomy | 0.32 (0.72) | 0.55 (0.93) | -0.29 |
| | | | Competence (mastery) | 0.32 (0.72) | 0.00 (0.00) | 0.54 |
| | | | Competence (structure) | 0.05 (0.21) | 0.00 (0.00) | 0.26 |
| | | | Relatedness | 0.41 (0.85) | 0.91 (1.45) | -0.46 |
| | 2 | Support | Autonomy | 1.29 (1.27) | 0.64 (0.92) | 0.56 |
| | | | Competence (mastery) | 0.14 (0.48) | 0.00 (0.00) | 0.37 |
| | | | Competence (structure) | 1.57 (1.08) | 1.55 (1.29) | 0.02 |
| | | | Relatedness | 3.19 (1.94) | 3.36 (1.75) | -0.09 |
| | | Thwart | Autonomy | 0.24 (0.70) | 0.00 (0.00) | 0.42 |
| | | | Competence (mastery) | 0.33 (0.80) | 0.36 (0.67) | -0.04 |
| | | | Competence (structure) | 0.00 (0.00) | 0.00 (0.00) | -- |
| | | | Relatedness | 0.43 (0.87) | 0.00 (0.00) | 0.60 |
